# Inhibition of Canonical NF-κB Signaling by a Small Molecule Targeting NEMO-Ubiquitin Interaction

**DOI:** 10.1038/srep18934

**Published:** 2016-01-07

**Authors:** Michelle Vincendeau, Kamyar Hadian, Ana C. Messias, Jara K. Brenke, Jenny Halander, Richard Griesbach, Ute Greczmiel, Arianna Bertossi, Ralf Stehle, Daniel Nagel, Katrin Demski, Hana Velvarska, Dierk Niessing, Arie Geerlof, Michael Sattler, Daniel Krappmann

**Affiliations:** 1 Research Unit Cellular Signal Integration, Helmholtz Zentrum München für Gesundheit und Umwelt, Ingolstädter Landstr. 1, 85764 Neuherberg, Germany; 2 Assay Development and Screening Platform, Institute of Molecular Toxicology and Pharmacology, Helmholtz Zentrum München für Gesundheit und Umwelt, Ingolstädter Landstr. 1, 85764 Neuherberg, Germany; 3 Institute of Structural Biology, Helmholtz Zentrum München für Gesundheit und Umwelt, Ingolstädter Landstr. 1, 85764 Neuherberg, Germany; 4 Center for Integrated Protein Science Munich at Biomolecular NMR Spectroscopy, Department Chemie, Technische Universität München, Lichtenbergstr. 4, 85747 Garching, Germany; 5 Biomedical Center of the Ludwig-Maximilians University München, Department of Cell Biology, Großhadener Str. 9, 82152 Planegg-Martinsried, Germany; 6 Protein Expression and Purification Facility, Institute of Structural Biology, Helmholtz Zentrum München für Gesundheit und Umwelt, Ingolstädter Landstr. 1, 85764 Neuherberg, Germany

## Abstract

The IκB kinase (IKK) complex acts as the gatekeeper of canonical NF-κB signaling, thereby regulating immunity, inflammation and cancer. It consists of the catalytic subunits IKKα and IKKβ and the regulatory subunit NEMO/IKKγ. Here, we show that the ubiquitin binding domain (UBAN) in NEMO is essential for IKK/NF-κB activation in response to TNFα, but not IL-1β stimulation. By screening a natural compound library we identified an anthraquinone derivative that acts as an inhibitor of NEMO-ubiquitin binding (iNUB). Using biochemical and NMR experiments we demonstrate that iNUB binds to NEMO_UBAN_ and competes for interaction with methionine-1-linked linear ubiquitin chains. iNUB inhibited NF-κB activation upon UBAN-dependent TNFα and TCR/CD28, but not UBAN-independent IL-1β stimulation. Moreover, iNUB was selectively killing lymphoma cells that are addicted to chronic B-cell receptor triggered IKK/NF-κB activation. Thus, iNUB disrupts the NEMO-ubiquitin protein-protein interaction interface and thereby inhibits physiological and pathological NF-κB signaling.

NF-κB signaling plays a crucial role in inflammation, immune responses, survival and cell proliferation^1^. The canonical NF-κB signaling can be activated through various pathways including TNF receptor (TNFR), IL-1 receptor (IL-1R) or T-cell receptor (TCR)^2^,^3^. Upon ligand binding, proximal signaling events converge at the IκB kinase (IKK) complex, which acts as the gatekeeper of NF-κB signaling by phosphorylating and inactivating the inhibitors of NF-κB (IκBs).

The IKK complex consists of two catalytic subunits IKKα/IKKβ and the regulatory subunit IKKγ/NEMO (NF-κB essential modulator)^4^. The IKK regulatory subunit NEMO serves as a critical integrating platform that couples upstream receptor signaling complexes to the catalytic IKKs^5^,^6^. Biochemical and genetic studies have highlighted a pivotal function of poly-ubiquitination for IKK/NF-κB activation. It has been shown that IKK upstream signaling adaptors like RIP1 (TNFR), IRAK1/4 (IL-1R) or MALT1 (TCR) are modified by ubiquitin (Ub) chains to recruit NEMO and thereby promote IKK activation^2^,^7^,^8^. Assembly of Met1-linked (linear) Ub chains by the LUBAC (linear Ub assembly complex) is also essential for IKK/NF-κB activation in response to various stimuli^9^,^10^,^11^,^12^.

The Ub binding surface in NEMO called UBAN (Ub binding in ABIN and NEMO) has been co-crystallized with linear as well as with K63 Ub chains^13^,^14^. Despite the fact that the NEMO C-terminus comprising the UBAN and the zinc finger (ZF) domain has a high preference for binding to linear Ub chains, it can also bind K63 or K11 chains^15^,^16^,^17^,^18^. The importance of non-covalent NEMO-Ub interaction for delivering upstream signaling events in response to TNFR, IL-1R or TCR/BCR stimulation to activate the IKK complex has been highlighted by several studies^19^. Moreover, chronic B-cell receptor (BCR) signaling in the aggressive activated B-cell (ABC) subgroup of diffuse large B-cell lymphoma (DLBCL) has been shown to require regulatory ubiquitination and the LUBAC^20^,^21^. Overexpression of the NEMO_UBAN_ or cell permeable peptides comprising the UBAN or leuzine zipper (LZ) regions interfered with ubiquitin association or NEMO oligomerization and impeded NF-κB activation^22^,^23^,^24^. Thus, disruption of NEMO-ubiquitin binding may be a promising strategy to inhibit inducible physiological as well as chronic pathological NF-κB activation.

In this work, we demonstrate by direct comparative analyses that UBAN-dependent NEMO-ubiquitin interaction is required for NF-κB in response to TNFα, but not IL-1β stimulation. We identified an inhibitor of NEMO-Ub binding (iNUB), an anthraquinone derivative of the natural product Emodin, as a small molecule that binds to the NEMO_UBAN_. Using biochemical, biophysical and NMR experiments we show that iNUB acts as a protein-protein interaction (PPI) inhibitor by preventing the recruitment of linear Ub chains. Consistent with the genetic data, iNUB inhibits TNFα, but not IL-1β-triggered NF-κB signaling. iNUB interferes with antigen receptor signaling to NF-κB and acts toxic in human lymphoma cells that are addicted to chronic BCR signaling.

## Results

### Differential requirement of NEMO_UBAN_ for TNFα and IL-1β-induced NF-κB signaling

**Figure 1:**
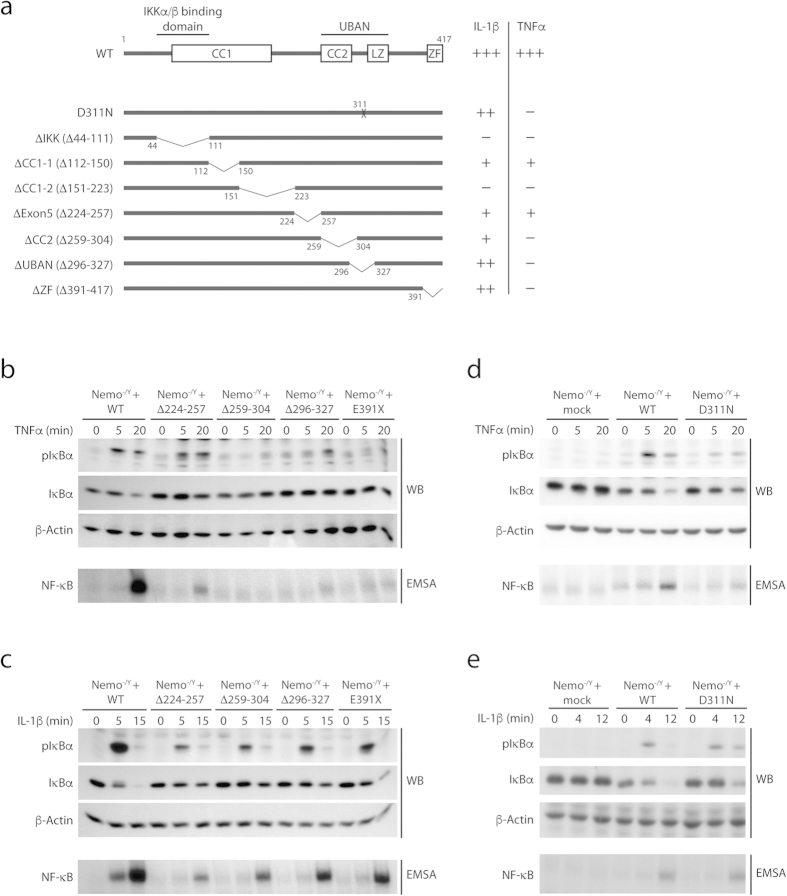
NEMO-Ub binding is essential for TNFα signaling, but not for IL-1β signaling. (**a**) Schematic illustration of NEMO deletions or point mutations. (**b**,**c**) Effects of NEMO C-terminal deletion mutants on TNFα and IL-1β signaling. NEMO^−/Y^ MEFs were reconstituted with mock, NEMO WT or different C-terminal deletion constructs and stimulated with TNFα (**b**) or IL-1β (**c**). Effects on NF-κB signaling were investigated by determining IκBα phosphorylation and degradation in Western Blots as well as NF-κB-DNA binding by EMSA. (**d**,**e**) Effects of NEMO D311N on TNFα and IL-1β signaling. NEMO^−/Y^ MEFs were reconstituted with mock, NEMO WT or NEMO D311N point mutant and stimulated with TNFα (**d**) or IL-1β (**e**). Analysis on NF-κB signaling were performed as in (**b**,**c**).

The critical role for the binding of Ub chains to the NEMO_UBAN_ motif has been demonstrated by multiple NEMO mutations that either directly prevent NEMO-Ub binding or indirectly disturb the structural conformation of the coiled-coil2/leucine-zipper (CC2-LZ) region^13^,^17^,^25^. Mutations in NEMO were shown to impair IKK/NF-κB activation after TNFα and IL-1β stimulation^13^,^26^. However, the requirement of the UBAN and other regions in NEMO for TNFα and IL-1β-induced NF-κB signaling has not been directly compared. We used NEMO deficient mouse embryonic fibroblasts (NEMO^−/Y^ MEFs) that were reconstituted with NEMO WT or NEMO mutants (Fig. 1a). The co-expressed surface marker human ΔCD2 was used for sorting homogenous populations of infected cells (>90%) that expressed NEMO proteins at equivalent levels (Supplementary Fig. 1a–d)^18^. Whereas NEMO WT was able to efficiently rescue defective NF-κB signaling in response to TNFα or IL-1β stimulation as reflected by IκBα phosphorylation and degradation, as well as activation of NF-κB DNA binding, deletion of the IKK interaction surface (ΔIKK; Δ44–111) abolished NF-κB signaling (Supplementary Fig. 1e–g)^27^. The deletions ΔCC1-1 (Δ112–150) and ΔCC1-2 (Δ151–223) in NEMO did not interfere with IKKα/β association and led to a partial or complete inhibition of both TNFα and IL-1β signaling to NF-κB, respectively (Supplementary Fig. 1e–g). Similarly, deletion of the linker region between CC1 and CC2 encoded by exon5 (Δ224–257) reduced NF-κB activation in response to both inducers (Fig. 1b,c). In contrast to these mutations, removal of the CC2 (Δ259–304) or the Ub binding region in the UBAN (Δ296–327) prevented NF-κB signaling in response to TNFα, but only partially diminished IL-1β-triggered NF-κB activation (Fig. 1b,c). Moreover, the NEMO_UBAN_ point mutation D311N or the deletion of the C-terminal zinc finger (E391X: Δ391–417), two patient derived mutations that cause anhidrotic ectodermal dysplasia with immunodeficiency (EDA-ID), abolished TNFα-induced NF-κB signaling, but only mildly affected IL-1β stimulated NF-κB activation (Fig. 1d,e). Thus, while the NEMO CC2, UBAN and ZF in the C-terminus are essential for TNFα signaling, these domains are largely dispensable for IL-1β signaling, emphasizing that distinct NEMO surfaces are required for bridging the different receptor pathways to canonical NF-κB.

### Structural characterization of the NEMO_UBAN_ dimer and linear Ub_2_ in solution

To explore the possibility of small molecule inhibition to interfere with protein-protein interaction (PPI) of NEMO_UBAN_ and ubiquitin, we first addressed the stoichiometry and conformation of NEMO_UBAN_ alone and in complex with linear Ub_2_ (linUb_2_) in solution. Whereas a 2:2 stoichiometry had been reported for the NEMO_UBAN_:linUb_2_ complex in co-crystals (PDB 2ZVO)^13^, a 2:1 stoichiometry was described in solution^25^. Indeed, by size exclusion chromatography (SEC) in combination with static light scattering (SLS) we found that one NEMO_UBAN_ dimer associates with one linear Ub_2_, revealing a 2:1 stoichiometry for the complex (Supplementary Fig. 2a,b).

**Figure 2:**
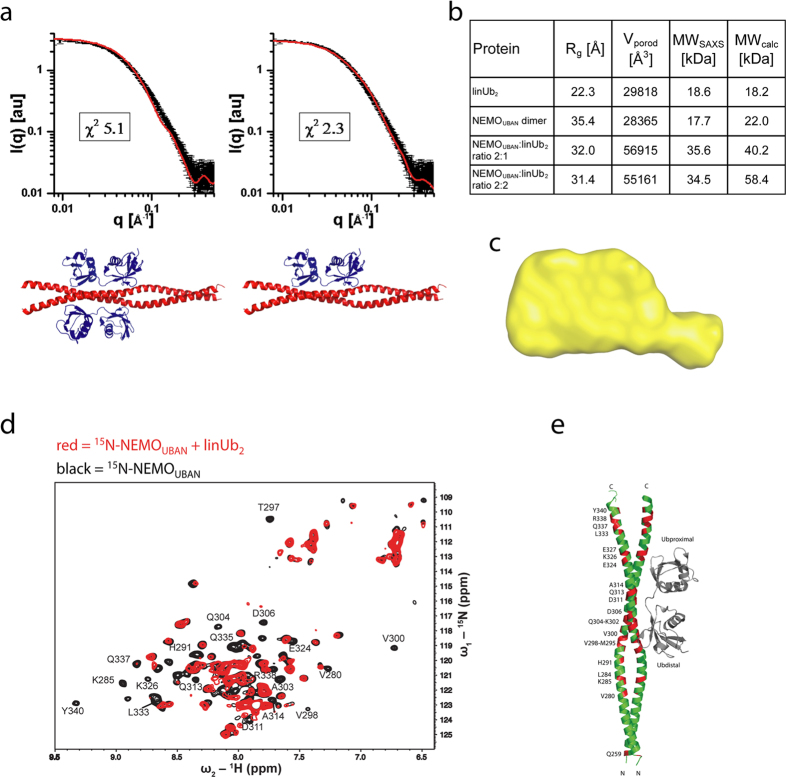
The NEMO-Ub_2_ complex has a 2:1 stoichiometry in solution. (**a**) The 2:1 stoichiometry of the NEMO-Ub_2_ complex is supported by comparison of the SAXS experimental curves with theoretical curves back-calculated from either the 2:2 NEMO-Ub_2_ co-crystal structure (PDB 2ZVO, 2:2) or a 2:1 complex, where one Ub_2_ was removed from the crystal structure. (**b**) The calculated MWs in the table show that independent of the mixing ratio of NEMO_UBAN_ and Ub_2_ a 2:1 complex is formed, and (**c**) the 2:1 ratio is further supported by the shape of the envelope of *ab initio* modeling with DAMMIF (shown in yellow). (**d**) NMR analysis of the NEMO_UBAN_-linUb_2_ interaction. ^1^H,^15^N TROSY NMR spectrum of 100 μM ^15^N-labeled NEMO in the absence (black) or presence of Ub_2_ (red) in a 2:1 (NEMO_UBAN_:Ub_2_) ratio. Amides, which disappear upon Ub_2_ addition are indicated. (**e**) Mapping of the backbone amides, which disappear upon linUb_2_ addition in panel d onto the NEMO_UBAN_ surface from the crystal structure (PDB 2ZVO).

To gain further insight into the structure of the NEMO_UBAN_:linUb_2_ complex, we performed small angle X-ray scattering (SAXS) experiments using NEMO_UBAN_ C347S (258–350). Microscale Thermophoresis (MST) and Isothermal Titration Calorimetry (ITC) measurements confirmed that the C347S mutation in NEMO does not affect the affinity (*K*_D_ ~ 1–2 μM) and the 2:1 stoichiometry of the NEMO_UBAN_:linUb_2_ complex (Supplementary Fig. 2c,d)^18^,^25^. SAXS data also revealed that NEMO_UBAN_ forms a dimer in solution (Supplementary Fig. 2e and 2b). However, the relatively high χ2 value indicates that the solution conformation of NEMO_UBAN_ is slightly different from the crystal structure (PDB 3FX0)^25^, suggesting that NEMO_UBAN_ in solution is not strictly linear. Comparison of the SAXS experimental curves for Ub_2_ with the theoretical curves back-calculated from the two published crystal structures for linear Ub_2_ suggests that the solution conformation of linear Ub_2_ is better represented by an ensemble of an open (PDB 3AXC)^28^ and a compact (PDB 2W9N)^29^ domain arrangements seen in previous crystal structures with 38%:62% population (Supplementary Fig. 2f). Calculated Porod volumes and MWs from the scattering curves obtained for solutions containing NEMO_UBAN_ C347S and linear Ub_2_ at ratios of 2:1 and 2:2 (Fig. 2a,b) are consistent with a complex of two NEMO_UBAN_ molecules (i.e. one NEMO dimer) bound to one linear Ub_2_. A DAMMIF model derived from the SAXS data of the 2:1 NEMO_UBAN_:Ub_2_ solution showed an elongated surface with only one hump consistent with an NEMO dimer bound to one Ub_2_ in solution (Fig. 2c).

We used Nuclear Magnetic Resonance (NMR) experiments to analyze the NEMO_UBAN_ binding interface to linear Ub_2_ in solution and compared it with the previously reported crystal structure^13^. We could assign 84% of the backbone NMR signals of NEMO_UBAN_ (258–350) and analyzed spectral changes induced upon the addition of unlabeled linear Ub_2_ in a 2:1 ratio (NEMO_UBAN_:Ub_2_) (Fig. 2d). Chemical shift perturbations were observed for multiple amino acids on NEMO that are directly contacting linear Ub_2_ in the crystal, e.g. V300, A303, Q304, D311, E324, E320. However, additional shifts were obtained for several residues distributed across the entire CC2-LZ region of NEMO that are not directly involved in contacting linear Ub_2_ (Fig. 2e). Taken together, the SAXS and NMR data suggest that, in solution, binding of linear Ub_2_ to NEMO_UBAN_ induces allosteric effects that modulate the overall structure and dynamics of the NEMO_CC2-LZ_ dimer. Such allosteric effects could also explain that only an asymmetric 2:1 complex is formed as allosteric changes induced by binding of one Ub_2_ molecule may prevent binding of a second Ub_2_ moiety on the opposite surface.

### Identification of NEMO-Ub binding inhibitors from a natural compound library

Due to the relatively low binding affinity of NEMO_UBAN_ to ubiquitin (*K*_D_ 1–2 μM for linear and 12–130 μM for K63 Ub_2_)^13^,^15^,^18^,^25^, the conformational dynamics of the NEMO_UBAN_ (see Fig. 2) and the selective requirement of the NEMO_UBAN_ for TNFα, but not IL-1β signaling to NF-κB (see Fig. 1), we wanted to test whether NEMO-Ub interaction could be susceptible to small molecule inhibition. We used the previously established ‘Dissociation-Enhanced Lanthanide Fluorescent Immunoassay’ (DELFIA) technology to detect binding of recombinant Myc-NEMO_UBAN_-StrepTagII (aa 246–350) and His-linUb_2_ in a multi-plate format (Supplementary Fig. 3a)^18^. As expected, linear Ub_2_ bound to NEMO_UBAN_ and association was abrogated by the UBAN mutation D311N (Supplementary Fig. 3b). We screened a small molecule library of 320 purified natural products for their ability to inhibit binding of linUb_2_ to NEMO_UBAN_ (Supplementary Fig. 3c). The screening provided robust signal to noise ratio (Z’ factor 0.78) and delivered 4 hits, which significantly reduced the signal for NEMO-Ub binding below 50% (Supplementary Fig. 3d). One of the compounds that most severely reduced the signal was a derivative of the anthraquinone Aloe-Emodin. Anthraquinones like Emodin and Aloe-Emodin are natural products, found as active ingredients in Chinese herbs, with demonstrated multiple biological effects including anti-inflammatory and anti-cancer activities^30^. Since Emodin affects several molecular pathways including NF-κB signaling^30^, we wanted to test, if distinct anthraquinones could affect the association of NEMO and ubiquitin. We tested the Aloe-Emodin derivative, Emodin as well as 10 other chemically synthesized anthraquinones for their ability to inhibit the interaction of NEMO and linear Ub_2_ in DELFIA (Supplementary Fig. 3e). Interestingly, whereas most anthraquinones including Emodin did not significantly inhibit NEMO-Ub_2_ binding, Aloe-Emodin derivative and anthraquinone 1 (8-hydroxy-9,10-dioxo-9,10-dihydro-1-anthracenyl 2-phenylcyclopropanecarboxylate) were able to significantly inhibit this interaction.

### iNUB disrupts NEMO-Ub interaction by binding to NEMO_UBAN_

**Figure 3:**
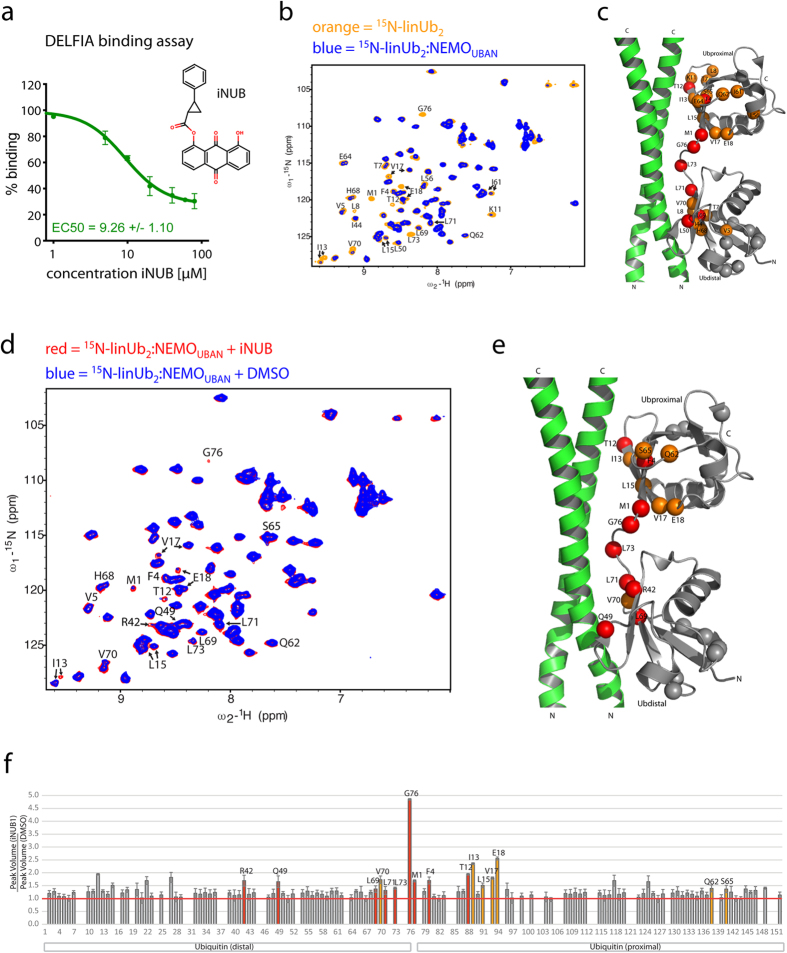
The small molecule iNUB inhibits binding of NEMO_UBAN_ to linUb_2_. (**a**) DELFIA assay detection of StrepTagII-NEMO_UBAN_ (242–350) binding to His-tagged linUb_2_ with or without iNUB. Increasing iNUB concentrations disrupted binding of NEMO to linUb_2_ binding with an EC_50_ of 9.26 μM. (**b**) NMR analysis of the NEMO_UBAN_-linUb_2_ interaction. ^1^H, ^15^N HSQC spectra of 100 μM ^15^N-labeled linUb_2_ in absence (orange) and in presence of unlabeled NEMO_UBAN_ C347S (258–350) (blue) at 1:1 stoichiometry. (**c**) Backbone amide resonances of Ub_2_ resonances showing strongest changes upon NEMO_UBAN_ addition are annotated and indicated as spheres on the NEMO crystal structure (PDB 2ZVO). Red spheres indicate residues with unambiguous chemical shift assignments, while orange color shows amides, which could not be unambiguously assigned to the proximal or distal Ub moieties or showing signal overlap. Gray spheres are the amides of the corresponding residues in the other Ub module. (**d**) ^1^H, ^15^N HSQC spectra of 100 μM ^15^N-labeled Ub_2_ bound to the NEMO_UBAN_ (1:1) in the presence of DMSO (blue) or 290 μM iNUB (red). NMR signals of linUb_2_ residues that showed reduced signal intensity upon binding to NEMO_UBAN_ are partially restored upon treatment with iNUB. (**e**) Mapping of Ub residues that show strongest effects upon iNUB addition onto the NEMO_UBAN_ structure as in (**d**). (**f**) Ratio of amide peak volume upon iNUB versus DMSO addition for the ^15^N-labeled Ub_2_ complexed with unlabeled NEMO (1:1 stoichiometry).

To investigate the potential impact of distinct anthraquinones on NEMO-Ub binding, we determined the effects of the most effective inhibitor, i.e. anthraquinone 1, which we henceforth call inhibitor of NEMO-Ub binding (iNUB). In DELFIA, iNUB disrupted NEMO_UBAN_ interaction to linUb_2_ with an EC_50_ of 9.3 μM and even though it was not able to completely block the interaction, it reduced the signal more than 70% at 40–80 μM (Fig. 3a).

We used NMR titrations to confirm that iNUB was able to compete the binding of linUb_2_ to NEMO_UBAN_. NMR experiments recorded with ^15^N-labeled linUb_2_ in the absence (orange) or presence (blue) of unlabeled NEMO_UBAN_ C347S (Fig. 3b) show large spectral changes upon binding of NEMO (NEMO_UBAN_ : Ub_2_ 1:1). Consistent with a previous study (Fig. 3b)^25^, many Ub_2_ backbone amide NMR signals show a significant intensity reduction upon addition of NEMO_UBAN_, due to severe line broadening. Mapping of the Ub_2_ backbone amide resonances with strongest changes onto the crystal structure of NEMO-Ub_2_ (PDB 2ZVO) highlights the Ub_2_ interface on NEMO and the essential role of many Ub_2_ amino acids in contacting NEMO_UBAN_, which are confirmed by mutagenesis (Fig. 3c)^25^.

Next, we compared the chemical shifts of ^15^N-labeled linUb_2_ when bound to NEMO_UBAN_ (1:1) in the presence of DMSO or iNUB to test whether the addition of iNUB can compete with the NEMO-Ub_2_ interaction (Fig. 3d–f). Indeed, iNUB induced the reappearance of many linUb_2_ signals that were reduced upon NEMO binding. Notably, many of these residues have been previously shown to have a critical role in the NEMO_UBAN_ and Ub_2_ interaction based on the crystal structure and/or mutagenesis, e.g. R42, V70 and L73 in the distal Ub moiety and F4, T12 and I13 in the proximal Ub moiety^13^,^25^.

In addition, we determined the effects of Emodin, because many studies suggested that Emodin is able to impair NF-κB signaling^30^. Confirming our previous results, Emodin did not significantly affect NEMO-Ub_2_ binding up to 20 μM and slightly reduced binding by approximately 25% at 80 μM (Supplementary Fig. 4a). As expected, incubation of ^15^N-labeled linUb_2_-NEMO_UBAN_ complex (1:1) with Emodin resulted in fewer changes in chemical shifts of Ub_2_ amino acids, validating DELFIA results that Emodin exerts only a very mild effect on the NEMO-Ub interaction (Supplementary Fig. 4b–d).

**Figure 4:**
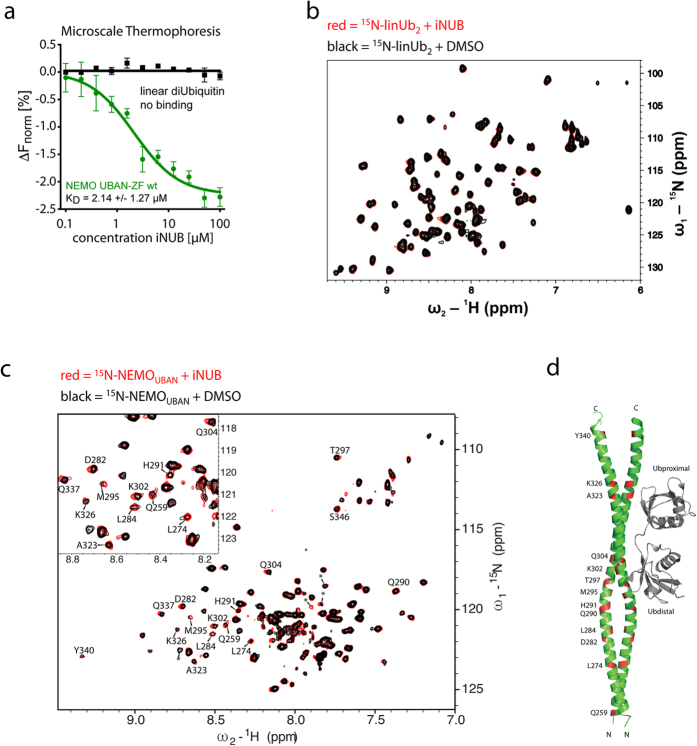
iNUB binds and alters the conformation of the NEMO_UBAN_. (**a**) Microscale Thermophoresis (MST) assays to determine binding of iNUB to NEMO_UBAN_ or linUb_2_. iNUB binds to Myc-NEMO_UBAN-ZF_-StrepTagII (242–350) with a *K*_D_ of 2.14 μM. (**b**) ^1^H,^15^N HSQC spectra of 50 μM ^15^N-labeled linUb_2_ in the absence (black) and presence of 1 mM iNUB (red). (**c**) NMR analysis of the NEMO_UBAN_-iNUB interaction. ^1^H,^15^N TROSY NMR spectrum of 117 μM ^15^N-labeled NEMO_UBAN_ (258–350) with DMSO (black) or 870 μM iNUB (red). Amide signals that are affected upon iNUB addition are indicated. Asterisks indicate unassigned NMR signals that are affected by iNUB. (**d**) Mapping of residues affected upon iNUB addition onto the NEMO_UBAN_ crystal structure (PDB 2ZVO).

To determine the mechanism of how iNUB disrupts NEMO-linUb_2_ interaction, we performed MST to elucidate to what component iNUB is binding (Fig. 4a). Titration of increasing iNUB concentrations to fluorescence-labeled NEMO_UBAN_ or linUb_2_ revealed that iNUB induced a change in thermophoresis only with NEMO_UBAN_. Thus, iNUB directly binds to NEMO with a *K*_D_ of ~2.14 μM, which is in the affinity range of NEMO-linUb_2_ interaction (Supplementary Fig. 2c,d)^25^. iNUB did not show any binding to linUb_2_ in MST assays. Consistent with these data, we did not detect significant chemical shifts on ^15^N-labeled linUb_2_ upon incubation with iNUB reflecting that iNUB is not binding to Ub_2_ (Fig. 4b).

We used ^15^N-labeled NEMO_UBAN_ WT (see Fig. 2) to verify a direct binding of iNUB to NEMO_UBAN_ by NMR (Fig. 4c). iNUB binding induced a number of changes in the NEMO_UBAN_ spectrum. We mapped the residues affected by iNUB incubation onto the crystal structure (Fig. 4d). A number of backbone amide NMR signals exhibit small chemical shifts and an increase in the intensity of NMR signals upon addition of iNUB. Similar to linUb_2_ addition (compare Fig. 2d,e), affected residues were not localized to one spot in the UBAN, but scattered throughout the NEMO_UBAN_ dimer. For some of the affected residues, a critical involvement in binding to ubiquitin or maintenance of the NEMO_UBAN_ coiled-coil structure has been demonstrated previously. For example, Q304 establishes hydrophobic interactions with V70 and L8 of the distal Ub_2_ moiety^13^. Q304 also forms a hydrogen bond to D306 that stabilizes the dimer and the mutation Q304A weakens NEMO-Ub_2_ interaction^17^,^25^. Furthermore, A323 is involved in preserving the α-helical structure and, the Incontinentia Pigmenti (IP) associated mutation, A323P completely distorts dimerization and thereby indirectly abrogates Ub binding^17^,^25^. Thus, just like binding of linUb_2_, iNUB alters the conformational dynamics of the NEMO_CC2-LZ_ and induces changes in amino acids within the NEMO_UBAN_ that are directly involved in binding to Ub and/or preserving the dimer interface. Thereby, iNUB can prevent the formation of the NEMO_UBAN_:linUb_2_ complex.

### iNUB inhibits UBAN-dependent NF-κB activation upon TNFα and TCR/CD28 stimulation

**Figure 5:**
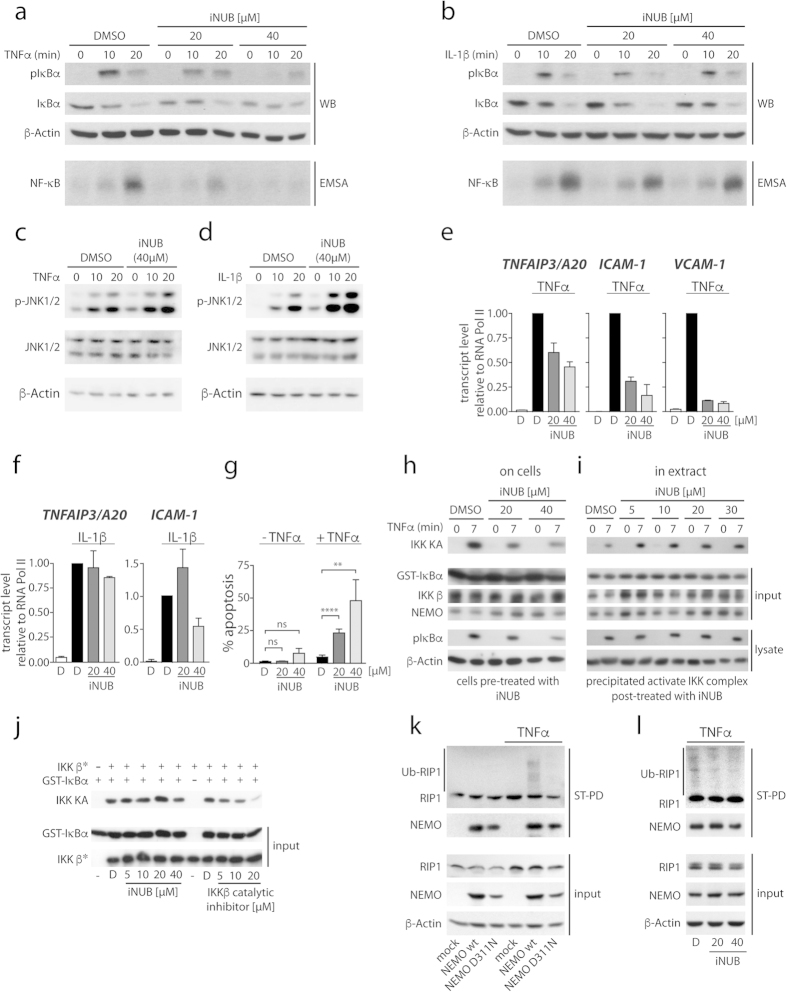
Inhibition of TNFα induced NF-κB signaling by iNUB. (**a**,**b**) Effects of iNUB on TNFα and IL-1β-induced NF-κB signaling. MEFs were treated with 20 μM and 40 μM iNUB or DMSO 6 h before stimulation with TNFα (**a**) or IL-1β (**b**). NF-κB signaling was investigated by determining IκBα phosphorylation and degradation in Western Blot and NF-κB activation by EMSA. (**c**,**d**) iNUB does not affect JNK activation. MEFs were treated with DMSO or iNUB (40 μM) 6 h prior to stimulation with TNFα (**c**) or IL-1β (**d**). Phosphorylation of JNK and total JNK were analyzed by Western Blotting. (**e**,**f**) iNUB inhibits NF-κB target gene expression after TNFα, but not IL-1β stimulation. MEFs were treated with 20 μM and 40 μM iNUB or DMSO and stimulated with TNFα (**e**) or IL-1β (**f**). mRNA was isolated and NF-κB target genes were investigated by qRT-PCR. (n = 3; +/− SD) (**g**) iNUB induces apoptosis after TNFα stimulation. MEFs were stimulated for 24 h in the presence or absence of iNUB. Apoptosis was analyzed by AnnexinV staining and FACS. (n = 3; +/− SD) (**h**) iNUB impairs TNFα-induced IKK activation. MEFs were treated with 20 μM and 40 μM iNUB before TNFα stimulation. IKK activity after NEMO IP was determined by *in vitro* kinase assay using GST-IκBα 1–72 as the substrate. (**i**,**j**) iNUB does not directly inhibit IKK activity. The cellular IKK complex after TNFα stimulation and following NEMO IP (**i**) or recombinant IKKβ (**j**) were treated with iNUB before *in vitro* kinase reaction. IKKβ inhibitor (SC-514) served as positive control. (**k**) TNFα dependent recruitment of NEMO to ubiquitinated RIP1 via UBAN. ST-PD of StrepTagII-NEMO WT and D311N expressing MEF. Co-precipitation of ubiquitinated RIP1 in unstimulated or TNFα stimulated cells was performed and analyzed by Western Blot. (**l**) iNUB impairs recruitment of NEMO to ubiquitinated RIP1. ST-PD was performed with StrepTagII-NEMO WT after TNFα stimulation in the presence and absence of iNUB. Co-precipitated ubiquitinated RIP1 was analyzed by Western Blot.

To test if iNUB is affecting NEMO_UBAN_ dependent NF-κB activation upon cell stimulation, we treated MEFs with 20 or 40 μM of iNUB before stimulation with TNFα or IL-1β (Fig. 5a,b). In the experimental setup, iNUB did not show cell toxicity up to concentrations of 60 μM as revealed in viability assays (Supplementary Fig. 5). Equivalent to the requirement for NEMO_UBAN_, iNUB reduced IκBα phosphorylation/degradation and NF-κB DNA binding in response to TNFα in a dose dependent manner, while it did not significantly affect IL-1β induced NF-κB signaling (Fig. 5a,b). To evaluate inhibitor selectivity, we determined activation of the MAPK JNK in MEFs in response to TNFα and IL-1β after iNUB treatment (Fig. 5c,d). Here, iNUB did not significantly affect TNFα or IL-1β stimulated JNK phosphorylation, showing that the compound is selectively acting on the NF-κB signaling pathway in response to TNFα.

Emodin, which was only mildly competing for NEMO-Ub binding *in vitro* also impaired IκBα phosphorylation/degradation as well as NF-κB DNA binding in response to TNFα, but not IL-1β stimulation (Supplementary Fig. 6a,b). However, cellular effects were considerably weaker when compared to iNUB treatment. Importantly, Emodin was also more selective in targeting TNFα triggered NF-κB signaling and IL-1β was not affected (Supplementary Fig. 6b).

We determined induction of NF-κB target gene activation after iNUB treatment (Fig. 5e,f). Whereas induction of *TNFAIP3/A20*, *ICAM-1* and *VCAM-1* transcripts were decreased by iNUB after TNFα stimulation (Fig. 5e), expression of *TNFAIP3/A20* and *ICAM-1* target genes upon IL-1β stimulation was not severely affected by iNUB (Fig. 5f). Also, Emodin impaired *TNFAIP3/A20*, *ICAM-1* and *VCAM-1* expression after TNFα stimulation (Supplementary Fig. 6c), but again to a lesser extent than iNUB. Congruent with the anti-apoptotic function of NF-κB, induction of apoptosis was elevated in TNFα treated cells either in the presence of iNUB or more weakly by incubation of Emodin, revealing that diminished NF-κB activation renders the cells more susceptible to TNFα-triggered cell death (Fig. 5g and Supplementary Fig. 6d).

Next, we investigated whether iNUB acts upstream of IKK activity in MEFs after TNFα stimulation (Fig. 5h,i). For this, we performed IKK kinase assays, after the cells have been treated with 20 or 40 μM of iNUB. The IKK complex was enriched by NEMO co-immunoprecipitation and IKK activity was measured in an *in vitro* kinase reaction. TNFα-induced IKK activation was markedly reduced in cells pre-treated with iNUB (Fig. 5h). To validate that iNUB is not acting directly on IKK kinases, but rather acts upstream on TNFα stimulation dependent IKK activation, we repeated the experiment in MEFs, but instead of treating the cells, we added iNUB in increasing concentrations to the precipitated IKK complex before the *in vitro* kinase reaction (Fig. 5i). In this case, iNUB was unable to inhibit IKK activity. Moreover, in contrast to a pharmacological IKKβ active site inhibitor, iNUB was not able to reduce activity of recombinant IKKβ in a pure *in vitro* kinase assay (Fig. 5j). Hence, inactivation of cellular IKK activity by iNUB is not due to a direct inhibition of IKK kinase activity, but rather a disturbed signaling upstream of the IKK complex in response to TNFα signaling.

To verify that iNUB affects NEMO UBAN dependent recruitment of Ub modified proteins in cells, we used NEMO^−/Y^ MEFs reconstituted with StrepTagII-NEMO WT or the UBAN mutant D311N (Fig. 5k). In agreement with previous results^31^, polyubiquitinated RIP1 was co-precipitated by StrepTactin pull-down (ST-PD) of NEMO WT after TNFα stimulation and association required an intact UBAN as it was lost in NEMO D311N expressing cells. In the same experimental setup, iNUB treatment (20 and 40 μM) of NEMO WT MEFs also inhibited association of NEMO to ubiquitinated RIP1 (Fig. 5l). Thus, similar to UBAN mutations, iNUB treatment prevented stimulation dependent recruitment of NEMO to ubiquitinated RIP1, suggesting that the inhibitor disconnects the IKK complex from upstream regulators.

**Figure 6:**
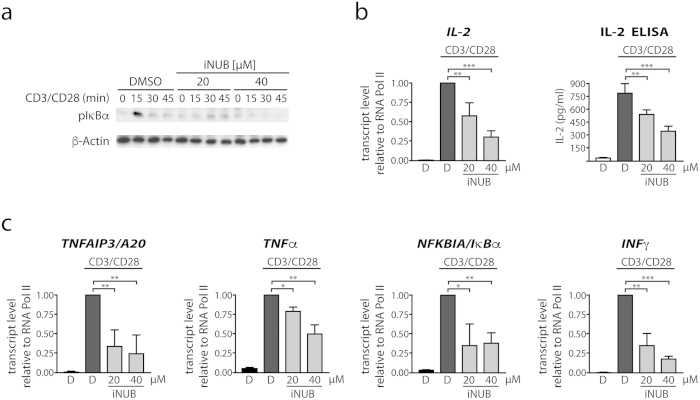
iNUB diminished NF-κB activation in murine T-cells after TCR/CD28 stimulation. (**a**) Inhibition of TCR/CD28 induced NF-κB signaling by iNUB. Primary murine CD4+ T-cells were incubated with iNUB (20 μM, 40 μM) or DMSO and co-stimulated with anti-CD3/anti-CD28 antibodies. IκBα phosphorylation was detected by Western Blotting. (**b**,**c**) iNUB inhibits NF-kB target gene expression in primary T-cells. CD4+ T-cells were treated as in (**a**,**b**) IL-2 expression was analyzed on mRNA level by qPCR after 3 h as well as on protein levels by ELISA after 20 h and (**c**) *TNFAIP3/A20*, *TNF*α, *NFKBAI/I*κ*B*α and *INFγ* mRNA expression was analyzed after 3 h (n = 3; +/− SD).

The NEMO_UBAN_ domain was also found to be essential for IKK/NF-κB activation in response to TCR/CD28 co-stimulation^18^. To determine if iNUB affects T-cell activation, we tested iNUB on NF-κB signaling and target gene expression in primary murine CD4+ T-cells stimulated with anti-CD3/CD28 antibodies (Fig. 6). Treatment with iNUB inhibited IκBα phosphorylation upon TCR/CD28 co-engagement of CD4+ T-cells (Fig. 6a). iNUB treatment led to a dose-dependent decrease in the induction of the NF-κB target genes *IL-2*, *TNFAIP3/A20*, *NFKBIA/I*κ*B*α and *IFNγ* (Fig. 6b,c). Induction of IL-2 secretion was also markedly reduced by iNUB as determined by ELISA (Fig. 6b). Thus, iNUB also impaired NEMO_UBAN_-dependent TCR/CD28 NF-κB signaling in primary CD4+ T-cells.

### iNUB is selectively toxic to IKK/NF-κB dependent ABC-DLBCL

**Figure 7:**
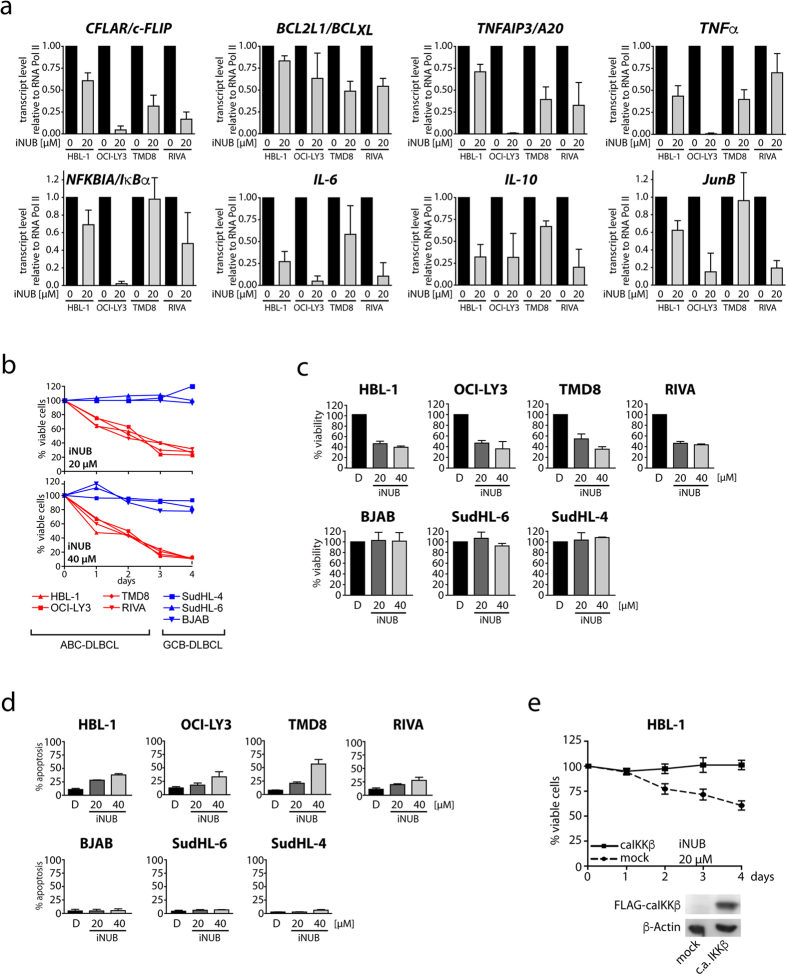
iNUB is toxic to ABC- but not GCB-DLBCL cells. (**a**) iNUB inhibits expression of NF-κB target genes in ABC-DLBCL cell lines. ABC-DLBCL cells (HBL-1, OCI-Ly3, TMD8, RIVA) were treated with iNUB (20 μM) for 24 h and NF-κB target genes were analyzed by qRT-PCR (n = 3; +/− SD). (**b**,**c**) Selective toxicity of iNUB in ABC- and GCB-DLBCL cells. ABC or GCB (SUDHL-4, SUDHL-6, BJAB) DLBCL cells were treated with single dose of 20 μM or 40 μM iNUB or DMSO. (**b**) Viable cells were counted after trypan blue exclusion over a period of 4 days (n = 4; optional representative experiment) or (**c**) MTT assay after 3 days of treatment (n = 4; +/− SD). (**d**) Apoptosis in ABC but not GCB-DLBCLs was induced by iNUB. Apoptotic cells were determined by Annexin-V staining of YO-PRO-3 negative cells and analyzed by FACS (n = 3; +/− SD). (**e**) Reduced viability of HBL-1 cells after iNUB treatment was rescued by expression of caIKKβ. HBL-1 cells expressing transduced with caIKKβ or mock were treated with iNUB (20 μM) and relative viable cells numbers was determined by trypan blue exclusion over 4 days. Expression of caIKKβ was determined by Western Blotting (n = 3; +/− SD).

The survival of ABC-DLBCL tumor cells relies on constitutive NF-κB activation driven by oncogenic mutations and/or chronic BCR signaling upstream of the IKK complex^32^. IKKβ inhibitors are well-documented to selectively kill ABC-DLBCLs^33^ and, thus we wanted to determine whether iNUB also affected pathological NF-κB signaling that results from uncontrolled antigen signaling in these lymphoma cells. NF-κB is necessary for maintenance of high expression level of anti-apoptotic and pro-inflammatory factors in ABC-DLBCL, and therefore we evaluated by qPCR the relative mRNA expression levels of a panel of NF-κB target genes (*c-FLIP*, *Bcl*_*XL*_, *TNFAIP3/A20*, *TNF*α, *NFKBIA/I*κ*B*α, *IL-6*, *IL-10* and *JunB*) in the ABC-DLBCL cell lines HBL-1, OCI-Ly3, TMD8 and RIVA (Fig. 7a). Despite some variations in the extent of inhibition of the different target genes, treatment of iNUB markedly reduced the expression of NF-κB regulated genes in ABC-DLBCL cells.

Since survival of ABC-DLBCL cells relies on constitutive NF-κB signaling, we determined the effects of 20 and 40 μM iNUB treatment on ABC-DLBCL survival by counting of viable cells. To monitor selectivity, we also measured viability in NF-κB independent germinal center B-cell (GCB) type of DLBCL cells lines (Fig. 7b). Whereas ABC-DLBCL cell lines HBL-1, OCI-Ly3, TMD8 and RIVA were sensitive to iNUB treatment and died in a time and dose-dependent manner, iNUB1 was not toxic to the GCB-DLBCL cells SudHL-4, SudHL-6 and BJAB. We confirmed the results by MTT assays as a second test for cellular viability (Fig. 7c). Again, only ABC-DLBCL cells but not GCB-DLBCL showed a dose-dependent induction of cell toxicity after iNUB treatment. Since NF-κB inhibition was shown to primarily enhance spontaneous apoptosis in ABC-DLBCL^33^, we determined iNUB dependent apoptosis. Indeed, apoptosis was increased by iNUB in ABC but not in GCB-DLBCL cells at 20 and 40 μM (Fig. 7d) again illustrating the selectivity of treatment. Finally, to verify that iNUB decreased viability of ABC-DLBCL by acting upstream of IKKβ, we lentivirally reconstituted HBL-1 cells with a constitutively active IKKβ (caIKKβ) mutant that drives canonical NF-κB signaling independent of upstream events^34^. While in mock infected HBL-1 cells the number of viable cells decreased by 40–50% after 4 days of iNUB treatment, expression of caIKKβ promoted a strong resistance to iNUB-induced cell death (Fig. 7e). Thus, iNUB selectively inhibited NF-κB activation in ABC-DLBCL and thereby counteracted cell survival and enhanced apoptosis.

## Discussion

Developing small molecule inhibitors that target PPIs is a challenging task, because the contact surfaces are usually flat and large (~1500–3000 Å^2^), contain many hydrophobic residues and largely lack distinct pockets for optimal small molecule binding^35^,^36^. In this study, we have demonstrated that PPI inhibition can be a novel small molecule strategy to inhibit IKK activation and thus canonical NF-κB signaling. We found that the anthraquinone derivative iNUB binds to the IKK regulatory subunit NEMO and, thereby alters NEMO dynamics to reduce its interaction with linear Ub_2_. In contrast to most PPI surfaces that have been targeted by small molecules, the NEMO-Ub binding interface does not contain distinct pockets, but is composed of extended α-helices forming a dimer. Interaction of the NEMO_UBAN_ and linUb_2_ is of relatively low affinity^25^, which can facilitate the disruption by small molecule compounds. Based on NMR, SAXS and biophysical analyses we propose that iNUB acts through modulating the structure and/or dynamics of the α-helical arrangement of the CC2-LZ region, as an unexpected and unique mode of action for small molecule inhibitors. We confirmed in cells that iNUB is acting on the level of the IKK complex, but not through direct targeting of catalytic IKKβ, as other conventional ATP-binding pocket inhibitors^37^. In line with the *in vitro* data on disrupted UBAN-Ub binding, iNUB inhibits UBAN dependent recruitment of NEMO to poly-ubiquitinated RIP1, a process that was shown to be critical for TNFα induced IKK/NF-κB activation^7^,^8^. iNUB did not inhibit activation of JNK, providing evidence that the compound is selectively impairing NF-κB signaling in response to TNFα. Further, congruent with the genetic data on the role of the UBAN, iNUB inhibited NF-κB signaling in response to TNFα and TCR/CD28 stimulation^18^, but did not affect UBAN-independent IL-1β stimulation (see also below). Also in ABC-DLBCL, a well-defined cancer entity whose survival relies on chronic activation of the BCR-IKK-NF-κB axis, iNUB inhibited NF-κB activity and selectively killed the tumor cells just like conventional IKKβ inhibitors^33^. Importantly, various studies have demonstrated the necessity of the LUBAC complex and linear ubiquitination for active BCR signaling^20^,^21^,^38^ thereby defining the molecular basis for iNUB inhibition in ABC-DLBCL.

The critical role of Ub binding to the NEMO_UBAN_ for IKK activation has been demonstrated by structural studies^13^,^14^,^17^,^25^. Moreover, inactivating point mutations within the UBAN and adjacent regions are causing IP in females and EDA-ID in males^39^, revealing that small alterations within this region exert strong effects on activation of NF-κB signaling. Indeed, we demonstrated by NMR that iNUB directly binds to the CC2-LZ region of NEMO and induces a number of chemical shift perturbations and resonance intensity changes. Some of these changes include residues that have been shown to be directly involved in Ub binding or NEMO dimerization^13^,^17^. However, other perturbations throughout the entire CC2-LZ region include residues in NEMO that are not expected to be involved in direct contacts with Ub_2_ and go far beyond the direct interactions mapped in the co-crystals^13^. Importantly, similar results were obtained when the interaction of linUb_2_ with NEMO_CC2-LZ_ was analyzed using NMR. These data suggest that many chemical shift perturbations observed are not caused by the direct binding of either iNUB or linUb_2_ to specific amino acids, but rather reflect that iNUB or linUb_2_ disturbs the α-helical structure and dynamics of the NEMO dimer to provoke these extensive changes. Moreover, the conformational alterations may also be important for activation of IKKβ^40^. Importantly, the structural and biophysical data suggest that iNUB is inducing similar, but less severe changes to the NEMO_CC2-LZ_ dimer as linUb_2_. Thus, iNUB seems to allosterically alter the structural conformation and dynamics of the NEMO_UBAN_ in a way that prevents its binding to linear Ub_2_.

Also, the widespread changes may explain the obvious discrepancy in the NEMO_UBAN_:Ub_2_ stoichiometry between the co-crystals and in solution assays. We confirmed the previously observed 2:1 stoichiometry of NEMO_UBAN_:Ub_2_ in solution^25^. Using SAXS data we could build a DAMMIF model showing a structure that best fits to the 2:1 NEMO stoichiometry of the NEMO_UBAN_:Ub_2_ complex. This complex is not symmetric as described for the crystal, but linUb_2_ is forming a hump and covering exclusively one side of the NEMO dimer. Together with the extensive spectral changes throughout the CC2-LZ seen in NMR, these data indicate that, in solution, binding of linUb_2_ to one side of the NEMO dimers precludes the interaction of a second Ub_2_ to the opposing surface. Thus, the 2:2 stoichiometry in the co-crystal may be induced by the crystal packing^13^.

In cells, iNUB is inhibiting TNFα, but not IL-1β driven NF-κB signaling. By direct comparison in a well-defined reconstitution system, we provide evidence that the NEMO_UBAN_ mutant D311N is required for NF-κB activation in response to TNFα, but largely dispensable for IL-1β stimulation. Moreover, even the removal of the UBAN or the C-terminal ZF domain of NEMO only partially impaired IL-1β signaling to NF-κB, despite the fact that it abrogated TNFα stimulation. In contrast, several N-terminal deletions resulted in equivalent decreases in NF-κB activation after TNFα or IL-1β treatment, ruling out a general variation within the experimental system. Given the pivotal role of Ub conjugation for IKK activation in all NF-κB signaling pathways, we were surprised to see such differences, and previous studies have reported decreased NF-κB activation in cells with NEMO_UBAN_ or NEMO_ZF_ mutations after IL-1β stimulation^13^,^26^,^41^,^42^,^43^. Zhang *et al.* reported severely reduced IKK activation after IL-1α stimulation in MEFs from NEMO^D311N/Y^ knock-in mice^26^. However, no direct comparison for the requirement of the NEMO_UBAN_ for NF-κB activation between TNF and IL-1 stimulation was performed. Interestingly, NF-κB signaling was reduced in fibroblasts from human EDA-ID patients carrying NEMO D311G mutations in response to TNFα and IL-1β, but in both cases there was still considerable NF-κB activation observed^41^. Thus, cell-specific and maybe even signal-specific (e.g. IL-1α versus IL-1β) variations may contribute to differential requirements of NEMO domains to trigger IKK/NF-κB in response to different stimuli. In fact, also the amount of NEMO is highly critical for signal propagation^18^. To minimize cell variability, we used reconstituted NEMO^−/Y^ MEFs and worked with FACS sorted cell pools that express equivalent NEMO amounts. Also other studies found fundamental differences in NEMO-dependent processes to NF-κB after TNFα and IL-1β stimulation. NEMO_UBAN_ displays a >100 fold higher affinity for M1 versus K63 Ub_2_^13^,^17^,^25^ and an Ub replacement strategy provided evidence for a requirement of K63-linked ubiquitination in IL-1β, but not for TNFα signaling^44^. In line with this, IL-1β-triggered recruitment of NEMO to supramolecular clusters required K63-linked Ub chains, whereas it did not for TNFα stimulation^45^. Also, cysteine-to-serine substitutions in the C-terminal ZF of NEMO have been shown to affect IKK activation in response to TNFα, but not IL-1β^46^. Even though we cannot fully resolve the discrepancies to some of the previous publications, our NEMO reconstitution provide clear evidence for differences in the requirement of C-terminal NEMO UBAN and ZF domains in the TNFα and IL-1β response. Congruent with our genetic complementation analyses, iNUB inhibited IKK/NF-κB activation and target gene expression in response to TNFα stimulation, but it did not affect IL-1β signaling in cells. Thus, small molecules that interfere with the binding of NEMO to Ub chains can be a strategy to selectively impair certain signaling pathways without affecting NF-κB response to other stimuli.

The potential of modulating Ub binding properties of the NEMO UBAN was previously shown by generating cell permeable peptides comprising the N-terminal part of the UBAN (UBI peptide)^22^. The UBI peptide contains the D311R, which impairs binding to Ub chains. However, mechanistically it was shown to bind to NEMO, thereby competing for Ub association and impairing NF-κB signaling after LPS or TNFα stimulation. Interestingly, in contrast to iNUB the UBI peptide only prevents association of K63-linked chains, while not affecting the binding of linear poly-Ub. Further, the steroidal lactone Withaferin A (WA) was shown to inhibit NF-κB signaling and to modulate NEMO-Ub binding properties of NEMO in cells and *in vitro*^47^,^48^. In contrast to iNUB, WA is covalently attached to the NEMO_ZF_, causing a gain-of-function that selectively enhances binding of NEMO to K48-linked Ub chains. However, how this impacts NF-κB signaling is currently unclear and it was also shown that WA can inactivate IKKβ directly by targeting Cys179 in the kinase domain^49^.

iNUB belongs to the class of anthraquinones that comprise natural products and the pharmacologically active ingredients of Chinese herbs. Most studied is the anthraquinone Emodin, whose anti-inflammatory and anti-cancer activity is well-established^30^. Emodin affects multiple pathways including NF-κB signaling, but the exact molecular targets are largely unresolved. Compared to iNUB, Emodin is a much weaker inhibitor of NEMO-Ub interaction and it is also less potent in inhibiting TNFα-triggered NF-κB activation and target gene expression. However, despite the mild effects on NF-κB signaling, Emodin still severely impacted on NF-κB target gene expression and apoptosis induction. Thus, our data indicate that Emodin may not primarily act on upstream signaling, but also seems to inhibit NF-κB activity further downstream, e.g. at the transcriptional level. Most likely also the pharmacological effects of iNUB will not only be restricted to prevention of NEMO-Ub binding, but we provide evidence that this mode of action contributes to NF-κB inhibition and downstream effects. Thus, our data provide a proof of concept that it is possible to dissect the cellular targets of these naturally-derived products and to optimize their activity, which may be of interest for future therapeutic approaches.

## Methods

### Reagents

TNFα (50435; Biomol Germany); IL-1β (211-11B; PeproTech); antibodies: anti-IκBα (sc-371, Santa Cruz, USA), anti-p-IκBα (9246; Cell Signaling), anti-NEMO (FL-419, Santa Cruz), anti-β Actin (sc-1616, Santa Cruz), anti-IKKβ (05–535; Millipore), anti-IKKα (14A231; Imgenex), anti-JNK1/2 (9252; Cell Signaling), anti-p-JNK1/2 (9251; Cell Signaling), anti-Rip1 (3493; Cell Signaling) anti-FLAG M2 (F3165, Sigma); anti-mCD3 (553057, BD Biosciences); anti-hCD28 (553294, BD Biosciences); anti-IgG (307-005-003, Dianova). Tris-d11 (CD4035P1, Cortecnet); DL-dithiothreitol-d10 (CD570P1, Cortecnet); D_2_O (151882, Aldrich); DMSO-d6 (156914, Aldrich); ^15^NH_4_Cl (CN80P100, Cortecnet); D-glucose ^13^C6 (CC860P20, Cortecnet); D-glucose ^13^C6-d7 (CCD860P20, Cortecnet).

### Cell Culture

DLBCL cell lines were cultured as previously described^50^. Mouse embryonic fibroblasts (MEF), NEMO^−/Y^ MEFs, reconstituted NEMO^−/Y^ MEFs and HeLa cells were cultured in DMEM supplemented with 10% FCS and 1% Pen/Strep. All cells were cultured in an H_2_O-saturated atmosphere with 5% CO_2_ at 37 °C.

### Stimulation and Western Blot

MEF cells were treated with the indicated amounts of iNUB or DMSO for 6 hours. Subsequently, MEF or HeLa cells were stimulated with 8 ng/ml TNFα or 1 ng/ml IL-1β. Murine primary T-cells were stimulated with mCD3/mCD28 (BD Biosciences) and hamster IgG antibodies (Dianova) on plates for 3 hours (qRT-PCR analysis) and 20 hours (ELISA analysis), respectively. DLBCL cell lines were treated with iNUB for 24h before cells were lysed for Western Blot analysis. Western blotting was carried out as described earlier^51^.

### Electrophoretic Mobility Shift Assay (EMSA)

For EMSA analysis, MEF cells were pretreated with iNUB or DMSO for 6 hours and stimulated with 8 ng/ml TNFα or 1 ng/ml IL-1β for several time points. EMSA was performed as described previously^52^. Briefly, cells were lysed and 2 μg of protein extract were incubated with a ^32^P-dATP–labeled, double-stranded NF-κB oligonucleotide probe (5′-CAGGGCTGGGGATTCCCCATCTCCACAGG-3′) or OCT1 oligonucleotide probe (5′-TGTCGAATGCAAATCACTAGAA-3′) and separated on native polyacrylamide gel electrophoresis before autoradiography.

### Production of NEMO for biophysical and structural studies

For screening, the NEMO_258–350_ construct was cloned, expressed and purified from *E. coli* strain BL21^18^. For other biochemical and biophysical measurements NEMO_258–350_ or NEMO_258–350_ C347S were cloned into pET vectors containing a 3C or TEV protease cleavable N-terminal His_6_-tags. Details on purification and removal of epitope tags by proteolytic cleavage are given in the Supporting Information.

### Microscale Thermophoresis (MST)

Recombinant proteins were produced as described earlier using *E. coli* strain BL21-CodonPlus (DE3) RILP (Stratagene) and the pIBA3plus expression system (IBA GmbH, Göttingen, Germany). MST assays were mainly carried out as previously described^18^. Serial dilutions of iNUB or Emodin were incubated with NT647-labeled NEMO_UBAN-ZF_ proteins for 30 minutes. MST assays were measured in a NanoTemper Monolith NT.115. Compound concentrations were plotted against percent changes of normalized fluorescence (Δ*F*_norm_ [%]), curve fitting was done with GraphPad Prism software and *K*_*D*_ values were determined.

### Small angle X-ray scattering (SAXS) analysis

SAXS measurements were performed on a Rigaku BIOSAXS1000 instrument with a HF007 microfocus generator equipped with a Cu-target at 40 kV and 30 mA. Transmissions were measured with a photodiode beamstop, q-calibration was made by an Ag-behenate measurement. Measurements were done in four 900 second frames, which were averaged. In these conditions no radiation damage was detected. Circular averaging and background subtraction was done with the Rigaku SAXSLab software v 3.0.1r1. Fits were made with crysol, ensembles with EOM, and distance distribution functions with GNOM, as provided with the ATSAS package v 2.5.0-2^53^. Molecular weights were calculated from the Porod Volumes. Three-dimensional *ab initio* models were generated using the DAMMIF software^53^.

SAXS measurements were made at 293 K with the following concentrations, NEMO_UBAN_ C347S four concentrations between 2.8 and 9.9 mg/ml, linear Ub_2_ five concentrations in the range of 0.9 to 9.9 mg/ml and three concentrations each for the 2:1 and the 2:2 complex in the range of 3.5 to 12.9 and 4.9 to 12.1 mg/ml respectively. The buffer contained 300 mM NaCl and 50 mM Tris-HCl at a pH of 8.0. No concentration dependent effects were detected. Agreement of SAXS theoretical curves back-calculated from published crystal structures to experimental curves was evaluated by χ2 values.

### NMR Spectroscopy

All NMR spectra were recorded on a Bruker AvanceIII 800 MHz spectrometer equipped with a TCI cryogenic or TXI room temperature probe head equipped with field gradient coils. All datasets were processed using NMRPipe^54^. Details for NMR spectroscopy are given in the Supplement.

### DELFIA Assay

Recombinant proteins were produced as described for MST assays. 20 pmol of StrepII-tagged NEMO proteins (UBAN domain) in DELFIA assay buffer (PerkinElmer) were bound to StrepTactin coated plates (IBA GmbH) and unbound proteins were removed after 2 h incubation by washing. Subsequently, bound NEMO proteins were incubated with several concentrations of iNUB or Emodin for 30 minutes before adding 125 pmol His-linUb_2_ in DELFIA assay buffer for further 2 h incubation. Again, unbound proteins were removed after the incubation time by washing. Finally, 500 ng/ml europium-labeled anti-His-tag antibody was added and incubated for 1 h. After extensive washing enhancer solution (PerkinElmer Life Sciences) was added and the signal was measured in a PerkinElmer Envision plate reader (excitation, 340 nm; emission, 615 nm).

### Viability, MTT and Apoptosis Assays

Viability, MTT (3-(4,5-dimethylthiazol-2-yl)-2,5-diphenyltetrazolium bromide) and Apoptosis Assays were carried out as described earlier^50^. Briefly, DLBCL cell lines were incubated with iNUB or DMSO in the indicated final concentrations for 24 hours. MEF cells were pretreated for 6 hours with iNUB or DMSO, respectively, before cells were stimulated with 8 ng/ml TNFα. Cell viability was analyzed by counting cells after trypan blue staining or by MTT cytotoxicity testing always in comparison to DMSO-treated control cells. Apoptosis rates were determined with Annexin-V-FITC staining of YO-PRO-3 negative cells (BD Pharmingen) after 24 hours of compound treatment in DLBCL and MEF cells. For FACS analysis an LSRII flow cytometer (BD) was used, and data were analyzed with FlowJo software (Treestar). Viability of mock (GFP) and caIKKβ-GFP expressing cells in response to iNUB treatment was determined by counting trypan blue stained cells.

### Lentiviral transduction

Cloning of hΔCD2-T2A-NEMO constructs into lentiviral vector, viral production, transduction of MEFs and sorting of hΔCD2 positive cells was performed as described previously^18^. Expression of constitutively active Flag-IKKβ (caIKKβ) mutant (SS176, 180EE) was conducted as described^50^. pLVTHM (Addgene plasmid # 12247), psPAX2 (Addgene plasmid # 12260) and pMD2.G (Addgene plasmid # 12259) were a gift from Didier Trono^55^.

### Quantitative qRT-PCR

cDNA generated from DNA-free RNA samples by reverse transcription was analyzed using LC-480 SybrGreen PCR mix (Roche) on a LC480 II Lightcycler system (Roche). 1 μg RNA was transcribed with Superscript II (Invitrogen) according to the manufacturer’s protocol, using random hexamers, with a final RNase H digestion step. The qRT-PCR experiments after TNFα stimulation were performed as described^18^, while qRT-PCR experiments in murine primary cells were carried out as described previously^50^. Quantification of NF-κB target genes were done with RNA PolII as control. All primer sequences are in the supplement.

### *In vitro* Kinase Assays

MEF cells were either pre-treated with iNUB before stimulation with TNFα or pre-stimulated with TNFα following a post-treatment with iNUB of the precipitated active IKK complex. In both cases IKKβ activity was investigated after immunoprecipitation (IP) of the IKK complex using an anti-NEMO antibody. Subsequently, recombinant GST-IκBα (aa 1–72) was added to the precipitated IKK complex and the activity was analyzed by detection of pIκBα in Western blot assays. Co-IP and Western blotting was done as described previously^52^. Recombinant active IKKβ was incubated with iNUB or an IKKβ inhibitor (sc-514, Santa Cruz) for 30 minutes. Subsequently, GST-IκBα was added and pIκBα was analyzed by Western Blotting.

### Detection of cellular interaction of NEMO and ubiquitinated RIP1

NEMO^−/Y^ MEF cells were transduced with StrepTagII-tagged constructs (mock, NEMO wildtype and NEMO D311N). Cells were grown in 150 mm cell culture plates until 90% confluence. Cells were either left untreated or treated with DMSO/iNUB for 6 hours and subsequently stimulated with 10ng/mL TNFα. Afterwards cells were lysed in 500 μl lysis buffer (25 mM HEPES pH 7.5, 150 mM NaCl, 0.2% NP-40, 10% glycerol, 1 mM DTT, 10 mM sodium fluoride, 8 mM β-glycerophosphate, 300 μM sodium vanadate and protease inhibitor cocktail) and StrepTactin pull-down (ST-PD) was performed by adding StrepTactin sepharose (IBA lifesciences) and incubating overnight. Co-precipitated RIP1 ubiquitination was detected in Western Blot analysis using RIP1 D94C12 antibody from cell signaling.

## Additional Information

**Accession codes:** The chemical shifts of NEMO and Ub_2_ are deposited in the BMRB, accession codes 26708 and 26709, respectively.

**How to cite this article**: Vincendeau, M. *et al.* Inhibition of Canonical NF-κB Signaling by a Small Molecule Targeting NEMO-Ubiquitin Interaction. *Sci. Rep.* **6**, 18934; doi: 10.1038/srep18934 (2016).

## Supplementary Material

Supplementary Information
