# Supplementary material for: Inhibition of Canonical NF-κB Signaling by a Small Molecule Targeting NEMO-Ubiquitin Interaction
Source: Sci Rep. 2016 Jan 7;6:18934. doi: 10.1038/srep18934 (PMC4703965; doi:10.1038/srep18934)

Supplementary Information

Inhibition of Canonical NF-κB Signaling by a Small Molecule Targeting NEMO-Ubiquitin Interaction

Michelle Vincendeau, Kamyar Hadian, Ana C. Messias, Jara K. Brenke, Jenny Halander, Richard Griesbach, Ute Greczmiel, Arianna Bertossi, Ralf Stehle, Daniel Nagel, Kathrin Demski, Hana Velvarska, Dierk Niessing, Arie Geerlof, Michael Sattler, and Daniel Krappmann

Supplementary Methods

Screening of small molecule library

Compound screening was performed using a natural compound library of 320 compounds at a concentration of 10 µM. This library was derived from Specs (specs.net, Netherlands) containing purified or synthesized natural products with a purity of >80%. The compounds were pre-selected by Specs with focus on diversity and a broad spectrum of natural sources, e.g. plants, fungi, bacteria, sea organisms. DELFIA assay for compound screening was performed as described above.

Compounds

Compounds from Chembridge/Hit2lead.com, USA: Anthraquinone 1 (=iNUB, 5109356), Anthraquinone 2 (5181426), Anthraquinone 3 (5212492), Anthraquinone 4 (5228191), Anthraquinone 5 (5253964), Anthraquinone 6 (5266859), Anthraquinone 7 (5631331), Anthraquinone 8 (5730401), Anthraquinone 9 (7259337), Anthraquinone 10 (7267603); other compounds: Emodin (7451.1, Roth, Germany); Aloe-Emodin derivative (AO-774/41465647, Specs, Netherlands

Production of NEMO for biophysical and structural studies

The constructs were transformed into *E. coli* strain BL21 (DE3) and cultured at 293 K in ZYM 5052 auto-induction medium[1]. Cells were lysed by sonication and His-tagged proteins purified by immobilized metal affinity chromatography (IMAC) using a 5-ml HiTrap Chelating HP column (GE Healthcare). The elution fractions were dialyzed overnight at 277 K in the presence of His-tagged 3C or TEV protease. The cleaved NEMO was further purified by IMAC and subjected to size exclusion chromatography using a HiLoad 16/60 Superdex 75 column

(GE Healthcare). The main elution peak containing NEMO was collected, concentrated to 10-110 mg/ml and stored at 277 K. A protease inhibitor cocktail (complete EDTA-free, Roche) was added to the final preparation according to the manufacturer's instructions in order to prevent degradation. Protein concentrations were determined by measuring the absorbance at 280nm using specific absorbance for NEMO 258-350 and 258-350 C347S of 0.271 mL/mg.

Protein production of labeled NEMO and Ub_2 for NMR studies was done by substituting the rich medium by M9 minimal medium with controlled ^{15}N and ^{13}C carbon sources. Production of triple labeled ^{2}H, ^{13}C ,^{15}N-NEMO for assignment was performed using M9 minimal medium, 100% D_2O and uniformly labeled ^{2}H,^{13}C-glucose. Purification of the proteins for NMR studies was carried out as described above for the unlabeled proteins.

NMR Spectroscopy

Assignments for linear Ub_2 were obtained by using HNCACB, ^{15}N-edited NOESY and ^{15}N-edited TOCSY 3D experiments[2]. Spectra were acquired using a 900 µM ^{13}C, ^{15}N-labeled linear Ub_2 sample at 290 K in 20 mM deuterated Tris-HCl pH 8.0, 50 mM NaCl, 10 % D_2O and at 298 K in 20 mM deuterated Tris-HCl pH 7.5, 50 mM NaCl, 10 % D_2O. Assignments of wild-type NEMO (258-350) were obtained using triple labeled samples in concentrations ranging from 850 µM to 1.2 mM based on data from 3D TROSY-based HNCA, HNCACB, HNCO, HN(CA)CO, HN(CA)CO and HN(CO)CACB with deuterium decoupling. Spectra were recorded at 308 K and sample conditions were 50 mM sodium phosphate pH 7.0, 50 mM NaCl and 10% D_2O. Some experiments were also repeated with $NEMO_{258\text{-}350}$ C347S. The software CARA (cara.nmr.ch) was used to assign linear Ub_2 and wild-type $NEMO_{UBAN}$.

Competition experiments were performed at 298 K using 100 µM of ^{15}N-labeled linear Ub_2 mixed in a 1:1 ratio with unlabeled $NEMO_{UBAN}$ C347S by adding the compounds (489.2 µM

Emodin, 290.7 μM iNUB, or DMSO-d6 for reference) and monitoring the changes by ^1H, ^{15}N-HSQC experiments (2048 (ω2) x 256 (ω1) time domain points and 64 scans). Sample conditions were 20 mM deuterated Tris-HCl pH 8.0, 50 mM NaCl, 0.3% Tween 20 and 10% D_2O. Due to poor compound solubility, some precipitation of the compounds was observed after the experiment. A comparison of peak volumes was made between the spectra of the complex (^{15}N-labeled linear Ub_2 and unlabeled $NEMO_{UBAN}$ C347S) with compound or deuterated DMSO added. Integration was performed using Sparky[3] with a Gaussian fit integration mode.

Binding of iNUB and Emodin to $NEMO_{UBAN}$ wild-type was performed at 308 K using 117 μM of ^{15}N-labeled NEMO in 50 mM deuterated Tris-HCl pH 8.0, 50 mM NaCl, 10mM DTT, 0.3% Tween 20 and 10% D_2O by adding the compounds to a final concentration of 870 μM, and monitoring the changes by ^1H, ^{15}N-TROSY experiments (1024k (ω2) x 128 (ω1) time domain points and 16 scans). A reference experiment was conducted in identical experimental conditions where the compound was replaced by an addition of the same volume of DMSO-d6.

Binding of iNUB and Emodin to Ub_2 was monitored at 298 K using 50 μM of ^{15}N-labeled Ub_2 in 20 mM deuterated Tris-HCl pH 8.0, 50 mM NaCl, 0.3% Tween 20 and 10% D_2O by adding the compounds to a final concentration of 1 mM and following changes by ^1H, ^{15}N-HSQC experiments (1024 (ω2) x 128 (ω1) time domain points and 8 scans). Reference experiments were conducted in identical experimental conditions where the compounds were replaced by addition of the same volume of DMSO-d6.

Titration of Ub_2 to $NEMO_{UBAN}$ was recorded at 308 K using 100 μM ^{15}N-labeled NEMO wild-type in 50 mM deuterated Tris-HCl pH 8, 50 mM NaCl, 10mM DTT, 0.3% Tween 20 and 10% D_2O by adding unlabeled Ub_2 781 μM solution to final molar ratios Ub_2:$NEMO_{UBAN}$ of 0, 0.06,

0.13, 0.20, 0.39, 0.42, 0.58 and 1.0 and following the changes by ^{1}H, ^{15}N-TROSY experiments (1024 (ω2) x 128 (ω1) time domain points and 32 scans).

Static Light Scattering (SLS)

Static light scattering (SLS) experiments of the different NEMO constructs, Ub_2 and the $NEMO_{UBAN}$:linUb_2 complex were performed at 303 K using a Viscotek TDA 305 triple array detector (Malvern Instruments) downstream to an Äkta Purifier (GE Healthcare) equipped with an analytical size exclusion column (Superdex 75 or 200 10/300 GL, GE Healthcare) at 277 K. The samples were run in 50mM Tris-HCl pH 8.0, 300mM NaCl, 0.01% (v/v) 1-thioglycerol with concentrations varying from 2.9-7.8 mg/ml at a flow rate of 0.5 ml/min. The molecular masses of the samples were calculated from the refractive index and right-angle light-scattering signals using Omnisec (Malvern Instruments). The SLS detector was calibrated with a 4 mg/ml BSA solution with 66.4 kDa for the BSA monomer and a *dn/dc* value of 0.185 ml/g for all protein samples.

Isothermal titration calorimetry

ITC measurements were carried out at 298 K using a ITC200 titration microcalorimeter (Microcal, GE Healthcare, NJ).. The calorimetric titration consisted of 32 injections of 0.4 µl of a 1.28 mM $NEMO_{UBAN}$ C347S sample, into the reaction cell containing 200 µl of 90 µM linear Ub_2, at a stirring speed of 1000 rpm. Sample conditions were 50 mM sodium phosphate pH 7.0 and 50 mM NaCl. The heat of dilution was obtained by titrating $NEMO_{UBAN}$ C347S into the sample cell containing only buffer and this was subsequently subtracted from each experimental titration. The ITC data were analyzed using the software Origin provided by Microcal.

Supplementary Table

Table qRT-PCR primers:

Target genes (murine)	FW Primer (5'-3')	Rev Primer (5'-3')
TNFAIP3/A20	GCTCAACTGGTGTCGTGAAG	ATGAGGCAGTTTCCATCACC
ICAM-1	GGAGACGCAGAGGACCTTAAC	CGCTCAGAAGAACCACCTTC
VCAM-1	AGTTGGGGATTCGGTTGTTCT	CCCCTCATTCCTTACCACCC
IL-2	GAGTGCCAATTCGATGATGAG	AGGGCTTGTTGAGATGATGC
TNFα	CCACCATCAAGGACTCAAATG	GAGACAGAGGCAACCTGACC
NFKBIA/IκBα	TTGCTGAGGCACTTCTGAAAG	TCTGCGTCAAGACTGCTACACT
INFγ	GCTCTGAGACAATGAACGCTAC	TCTTCCACATCTATGCCACTTG
β-actin	CCTCTATGCCAACACAGTGC	GTACTCCTGCTTGCTGATCC

Target genes (human)	FW Primer (5'-3')	Rev Primer (5'-3')
CFLAR/c-FLIP	TCAGAATCCTTTCCAGTGGG	TCCAGGCTTTCGGTTTCTTT
BCL2L1/BCL_{XL}	AAACTGGGTCGCATTGTGG	TCTCGGCTGCTGCATTGTTC
TNFAIP3/A20	TTTTGTACCCTTGGTGACCCTG	TTAGCTTCATCCAACTTTGCGG
TNFα	CCCAGGGACCTCTCTCTAATCA	GCTACAGGCTTGTCACTCGG
NFKBIA/IκBα	CCGCACCTCCACTCCATCC	ACATCAGCACCCAAGGACACC
IL-6	GGTACATCCTCGACGGCATCT	GTGCCTCTTTGCTGCTTTCAC
IL-10	CTGGAGGAGGTGATGCCCCAA	ACCTGCTCCACGGCCTTGCT
JunB	GCACTAAAATGGAACAGCCCTT	GGCTCGGTTTCAGGAGTTTG
RPII	GCACCACGTCCAATGACAT	GTGCGGCTGCTTCCATAA

Supplementary References

1 Studier, F. W. Protein production by auto-induction in high density shaking cultures. *Protein Expr Purif* **41**, 207-234 (2005).

2 Sattler, M., Schleucher, J. & Griesinger, C. Heteronuclear multidimensional NMR experiments for the structure determination of proteins in solution employing pulsed field gradients. *Prog NMR Spectrosc* **34**, 93-158 (1999).

3 Goddard, T. D. & Kneller, D. G. SPARKY3. *University of California, San Francisco*.

Supplementary Figure Legends

Supplementary Figure 1. Lentiviral transduction and analyses of NEMO proteins in MEFs.

(**a-b**) Transduction and sorting of human (h) ΔCD2-T2A-NEMO expressing MEFs. Exemplified sorting of infected MEFs transduced with NEMO Δ44-111 based on hΔCD2 surface expression (**a**). Sorting resulted in homogenous cell populations for all constructs that express hΔCD2 (>90 %) (**a and b**). (**c and d**) Expression levels of NEMO WT and mutant proteins after reconstitution in NEMO$^{-/Y}$ MEFs were analyzed by Western Blot and compared to MEFs from wildtype mice. (**e**) Co-IP of NEMO WT and three N-terminal deletion mutant was performed and analyzed for binding to IKKα and IKKβ by Western Blot. (**f and g**) Effects of NEMO N-terminal mutants on TNFα and IL-1β signaling. NEMO$^{-/Y}$ MEFs were reconstituted with mock, NEMO WT or different C-terminal deletion constructs and stimulated with TNFα (**f**) or IL-1β (**g**). Effects on NF-κB signaling were investigated by determining IκBα phosphorylation and degradation in Western Blots as well as NF-κB-DNA binding by EMSA.

Supplementary Figure 2. Analysis of NEMO and Ub_2 conformation and stoichiometry of the complex $NEMO_{UBAN}$:linUb$_2$.

(**a-b**) Determination of the molecular weight of $NEMO_{UBAN}$ C347S (green), Ub_2 (blue) and $NEMO_{UBAN}$;linUb$_2$ complex (red) using size exclusion chromatography in combination with static light scattering. The refractive index and right angle light scattering signals were monitored and used to determine the molecular weight (grey and black). 100 µl of the protein samples (2.9-7.8 mg/ml) were applied to a Superdex 200 10/300 GL column. NEMO was analyzed at 7.8 mg/ml and eluted as a homodimer of 21.6 kDa (calculated MW is 22.0

kDa), while Ub_2 (2.9 mg/ml) behaved as a monomer of 18.2 kDa (calculated MW is 18.2 kDa). NEMO and Ub_2 were mixed in a 2:1 molar ratio and 100 µl applied to a Superdex 200 10/300 GL column. Two peaks were obtained. The first peak eluted at 14.6 ml and a MW of 33.4 kDa. This peak contained a mixture of the $NEMO_{UBAN}$:linUb_2 complex and the remaining NEMO dimer. The second peak eluted at 17.3 ml contained the remaining Ub_2. Increasing the Ub_2:NEMO ratio let to an increase in the apparent molecular weight of NEMO (Insert in the chromatogram) to maximum of 37.3 kDa at a ratio of 12:1. As the k_{off} of the $NEMO_{UBAN}$:linUb_2 complex is relatively low, NEMO dimer and Ub_2 separate in the column, giving rise to a peak of NEMO dimer, which overlaps with the complex, leading to a lower apparent molecular weight (MW) of the complex (33.4 kDa at 2:1 NEMO:Ub_2). However, the apparent MW increases when the Ub_2:NEMO molar ratio is increased. (**c**) Interaction of $NEMO_{UBAN}$ C347S to Ub_2 was measured in MST assays. $NEMO_{UBAN}$ C347S binds Ub_2 with a K_D of 1.2 µM. (**d**) Isothermal titration calorimetry (ITC) measurement of the NEMO:Ub_2 interaction. NEMO C347S was titrated into the Ub_2 solution. The numbers show the fitting of the data, with N for stoichiometry, K_D for dissociation constant, ΔH for enthalpy change and -ΔS for entropy change. (**e**) Comparison of the SAXS experimental curve (black) with the theoretical curve back-calculated from the NEMO crystal structure (red; PDB 3FX0; construct 246-337) leads to a quite high χ2 value, indicating that the NEMO dimer crystal structure is considerably different than in solution. (**f**) Comparison of the SAXS experimental curves for Ub_2 (black) with the theoretical curves back-calculated from the two published crystal structures for linear Ub_2 PDB 2W9N (red) and PDB 3AXC (green), which do not fit to the experimental scattering well independently (right). An ensemble calculation with a 5 AlaSer flexible linker at the C-terminal part of the first Ub (residues 72-76) leads to a much better fit

(left). The resulting structures have the same domain distances as the published crystal data but different relative orientations of the Ub domains.

Supplementary Figure 3. Assay Development and Screening for NEMO-Ub$_2$ binding inhibitors.

(**a**) Schematic overview of the DELFIA assay using Strep-tagged coupled to Streptactin coated plates and His-tagged linear Ub$_2$. (**b**) DELFIA signal depends on intact UBAN. Signal by Myc-NEMO$_{UBAN}$-StrepTagII WT and His-linUb$_2$ was abolished by the UBAN mutation NEMO D311N. (**c**) Screening of a natural compound library using the DELFIA assay. Compound (10 µM) treated samples were compared to non-treated positive controls. 4 hits that inhibited the signal by >50% were identified. The overall Z' factor of the screening was 0.78. (**d**) Chemical structure of the 4 identified hits. (**e**) Effects of anthraquinone derivatives on NEMO$_{UBAN}$ binding to linear Ub$_2$. 12 anthraquinone derivatives comprising Aloe Emodin derivative and Emodin were tested in DELFIA binding assays at three concentrations.

Supplementary Figure 4. Effects of the natural compound Emodin on NEMO-Ub$_2$ binding.

(**a**) DELFIA assay detection of Myc-NEMO$_{UBAN}$-StrepTagII (242-350) binding to His tagged linear Ub$_2$ with or without Emodin. Graph shows the inhibition of NEMO-diUb binding by the addition of increasing Emodin concentrations. (**b**) NMR of linear Ub$_2$ in complex with NEMO$_{UBAN}$ in the presence of Emodin. ^{1}H, ^{15}N –HSQC spectrum of 100 µM ^{15}N-labeled Ub$_2$ with unlabeled NEMO$_{UBAN}$ (1:1 stoichiometry) in the presence of DMSO-d6 (blue) or 489.2 µM Emodin (red). Ub$_2$ backbone amides resonances, which changed upon treatment with Emodin are indicated. (**c**) Mapping of Ub$_2$ backbone amides perturbed upon Emodin addition in 'b' onto NEMO$_{UBAN}$ structure (PDB 2ZVO). Red spheres indicate peak shifts of

unambiguously assigned amino acids; orange spheres indicate peak shifts of ambiguously assigned or overlapping amino acids, while gray spheres represent the counterpart amino acid on the other Ub moiety. (**d**) Ratio of amide peak volume upon Emodin versus DMSO-d6 addition for the ^{15}N-labeled linUb$_2$ complexed with unlabeled NEMO (1:1 stoichiometry).

Supplementary Figure 5. iNUB Toxicity assessment in MEFs.

iNUB is not toxic to MEFs up to a concentration of 60 μM. Viability of MEFs was determined by MTT assay.

Supplementary Figure 6. Effects of Emodin on NF-κB signaling in MEFs.

(**a**) MEFs were treated with DMSO or Emodin (20 and 40 μM) for 6 h and subsequently stimulated with TNFα. NF-κB activation was determined by IκBα phosphorylation and degradation by Western Blot and NF-κB DNA binding by EMSA. (b) MEFs were treated with DMSO or Emodin as in a) prior to TNFα or IL-1β stimulation. NF-κB DNA binding activity was analyzed by EMSA and loading was controlled by OCT1 EMSA. (c) Effect of Emodin on NF-κB target gene expression after TNFα stimulation. MEFs were treated with 20μM and 40μM Emodin or DMSO and stimulated with TNFα. mRNA was isolated and NF-kB target genes were investigated by qRT-PCR. (n=3; +/- SD). (d) Emodin enhances apoptosis after TNFα stimulation. MEFs were stimulated for 24 h in the presence or absence of Emodin (20 and 40 μM) or DMSO. Apoptosis was analyzed by AnnexinV staining and FACS. (n=3; +/- SD).

Supplementary Figure 1

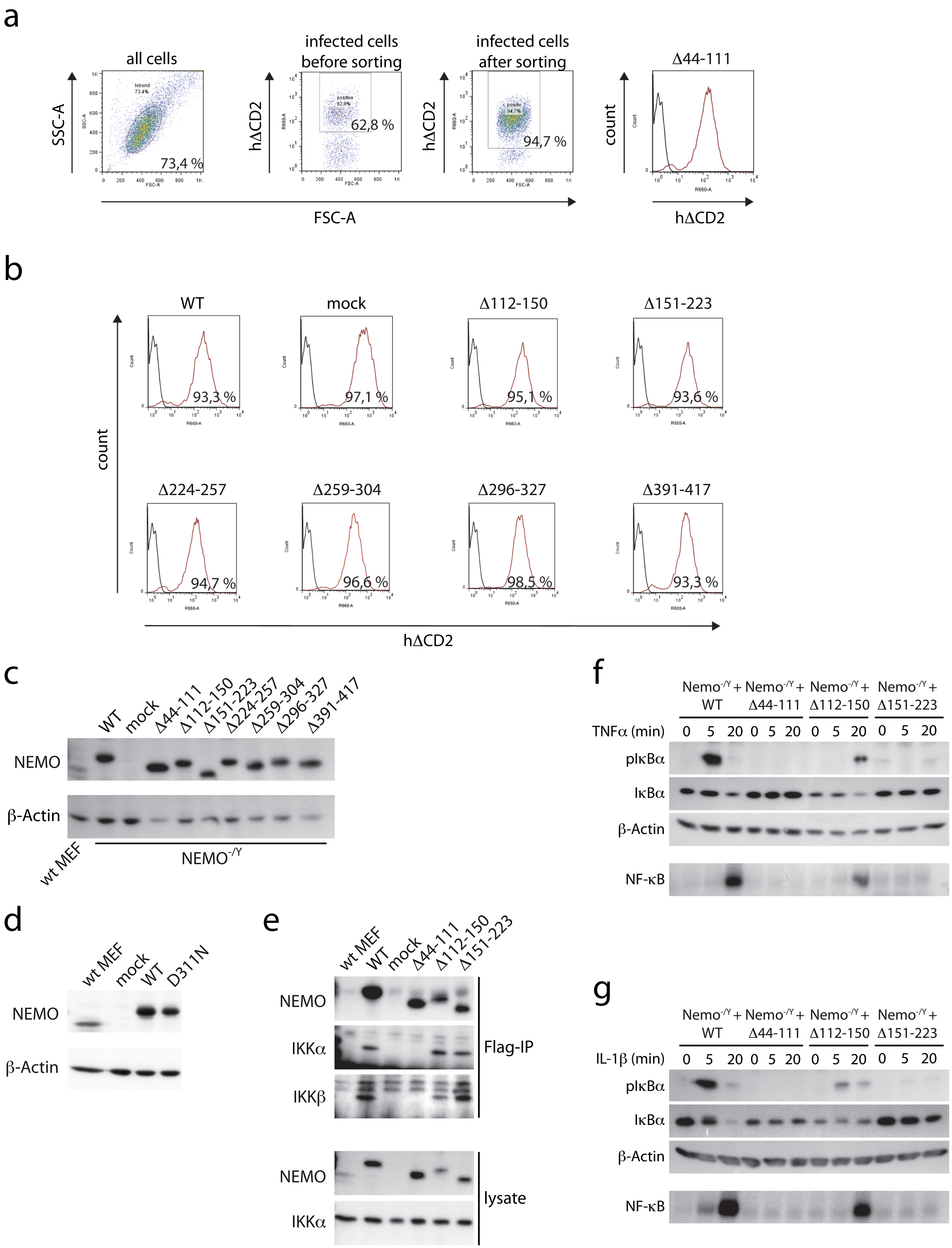

Supplementary Figure 2

a

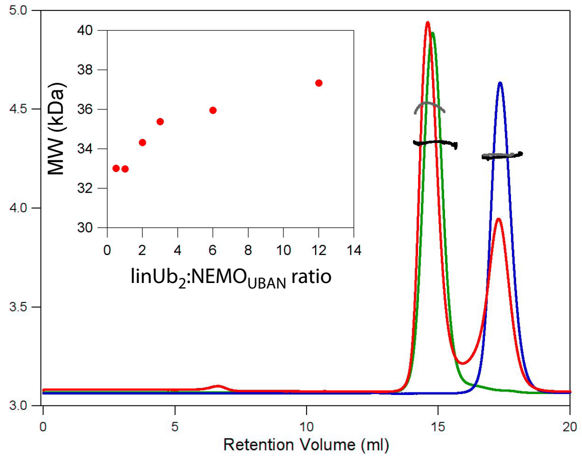

b

Protein	MW_{exp} [kDa]	MW_{calc} [kDa]
$NEMO_{UBAN}$ dimer	21.6	22.0
linUb2	18.2	18.2
$NEMO_{UBAN}$:$linUb_2$ ratio 2:1	33.4	40.2 $NEMO_{UBAN}$:$linUb_2$ (2:1)
$NEMO_{UBAN}$:$linUb_2$ ratio 2:12	37.3	40.2 $NEMO_{UBAN}$:$linUb_2$ (2:1)

c

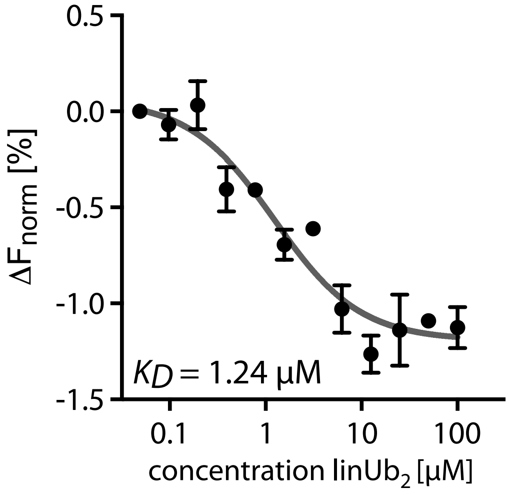

d

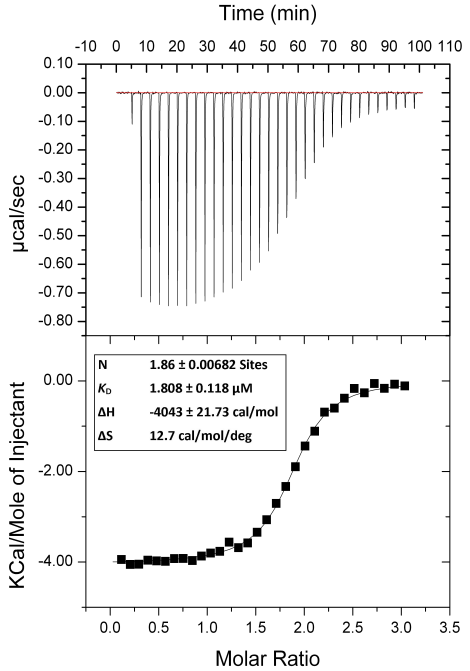

e

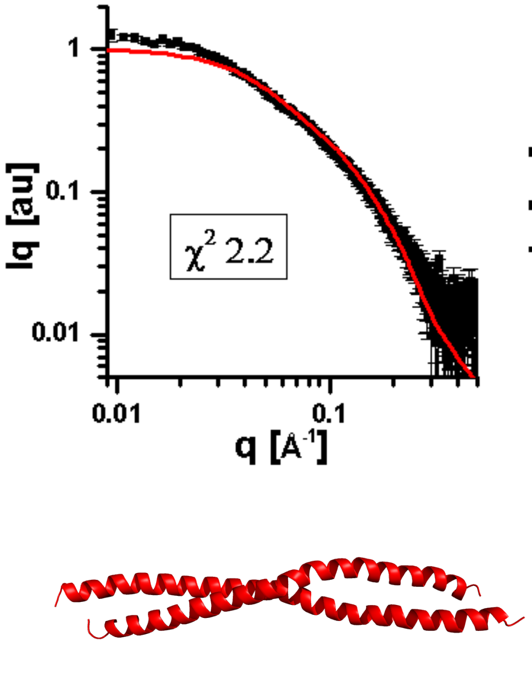

f

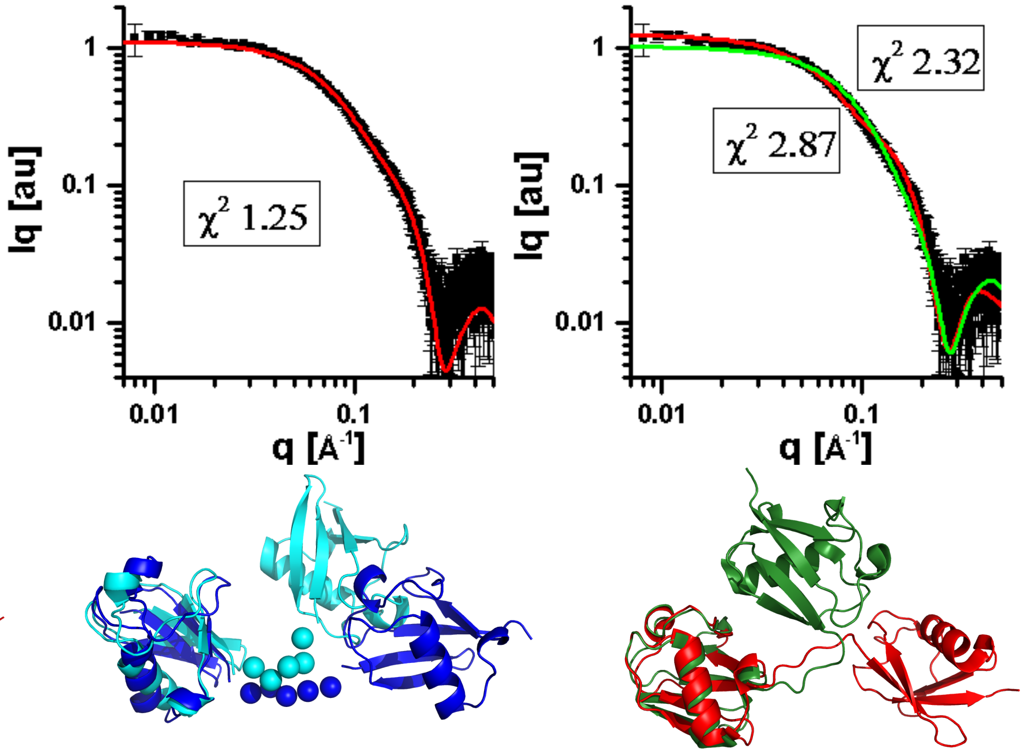

Supplementary Figure 3

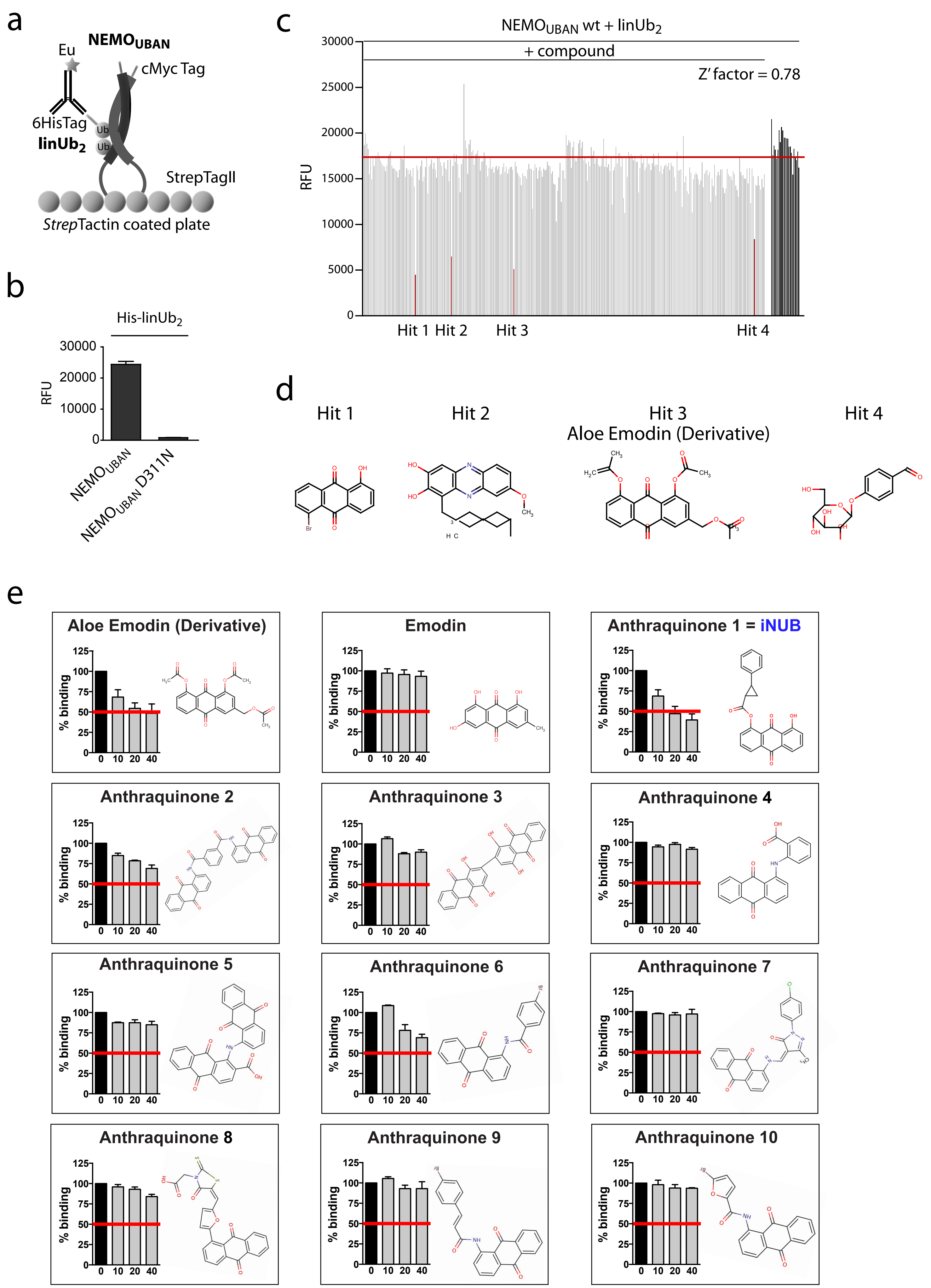

Supplementary Figure 4

a

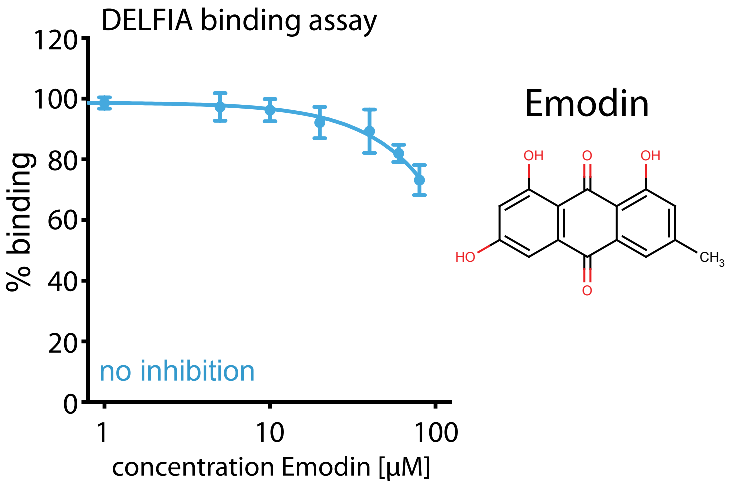

b

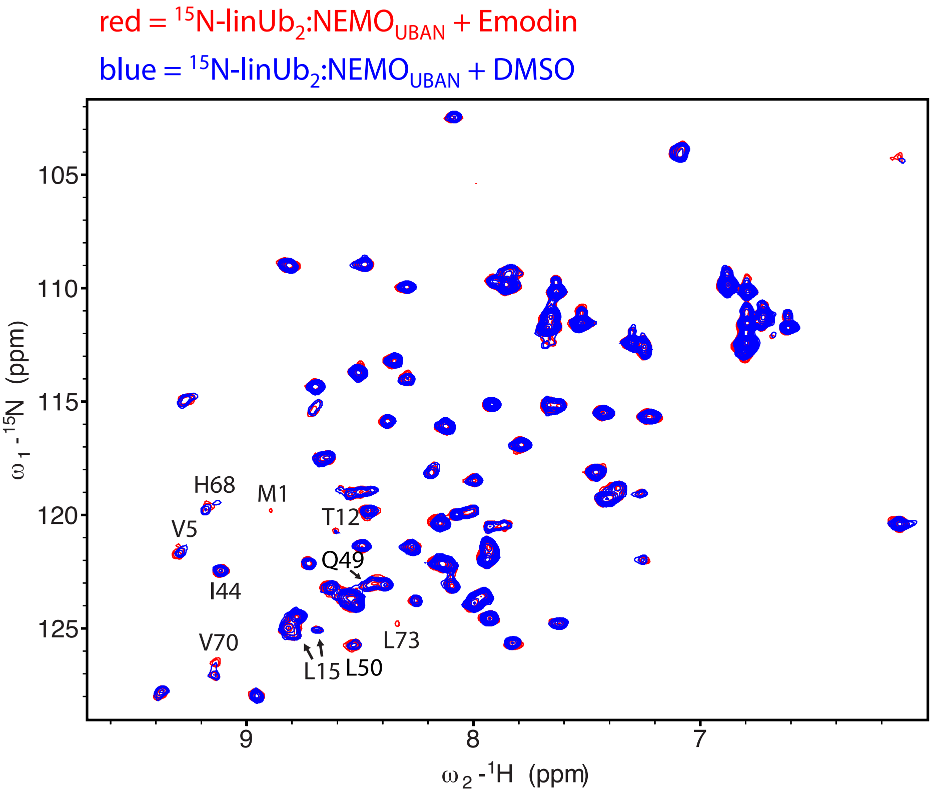

c

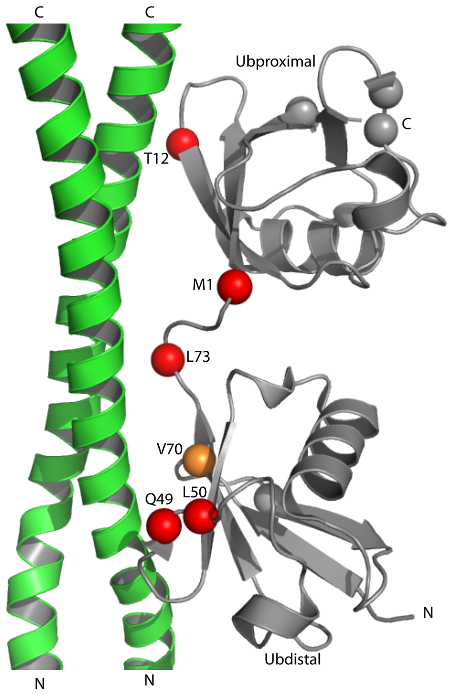

d

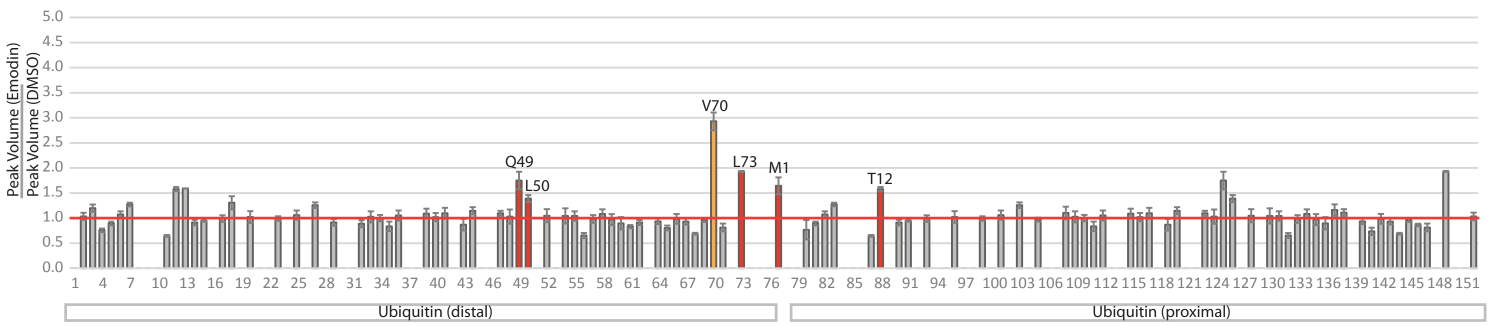

Supplementary Figure 5

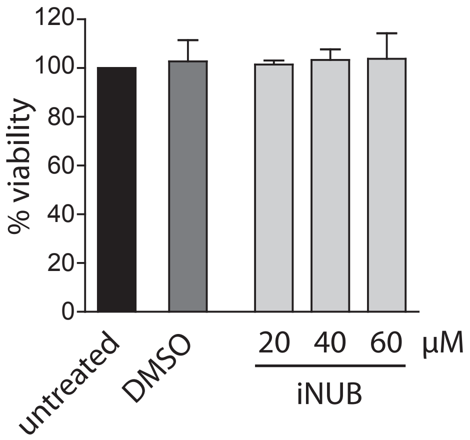

Supplementary Figure 6

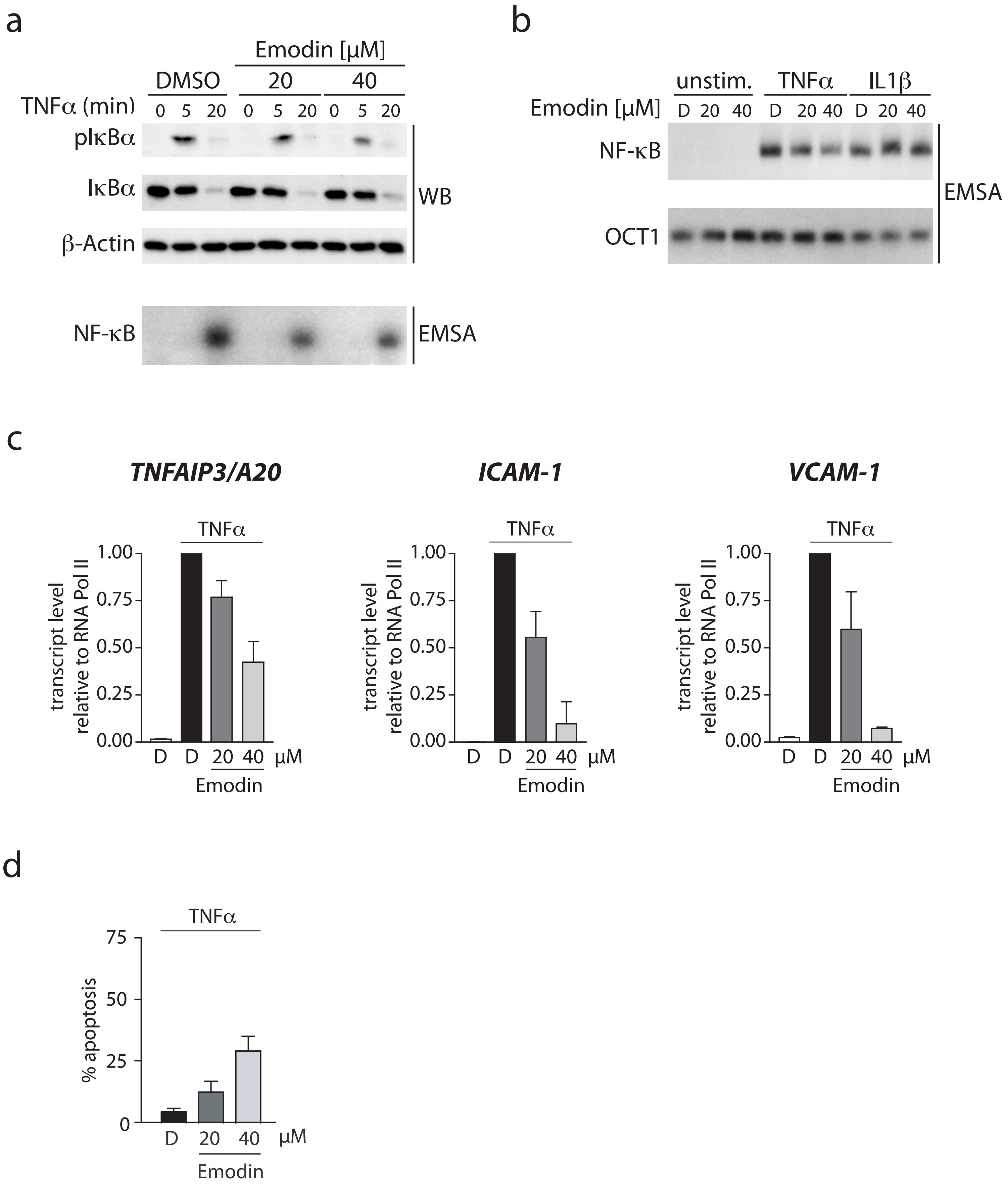

Supplement: Supplementary Information [file srep18934-s1.pdf]
